# The Eschenmoser coupling reaction under continuous-flow conditions

**DOI:** 10.3762/bjoc.7.135

**Published:** 2011-08-25

**Authors:** Sukhdeep Singh, J Michael Köhler, Andreas Schober, G Alexander Groß

**Affiliations:** 1 Institute for Chemistry and Biotechnology, Technische Universität Ilmenau, Weimarerstr. 32, D-98693-Ilmenau

**Keywords:** activation energy, episulfide, flow chemistry, keto imine, kinetics, S-alkylation, sulfide contraction, triisopropyl phosphite

## Abstract

The Eschenmoser coupling is a useful carbon–carbon bond forming reaction which has been used in various different synthesis strategies. The reaction proceeds smoothly if S-alkylated ternary thioamides or thiolactames are used. In the case of S-alkylated secondary thioamides or thiolactames, the Eschenmoser coupling needs prolonged reaction times and elevated temperatures to deliver valuable yields. We have used a flow chemistry system to promote the Eschenmoser coupling under enhanced reaction conditions in order to convert the demanding precursors such as S-alkylated secondary thioamides and thiolactames in an efficient way. Under pressurized reaction conditions at about 220 °C, the desired Eschenmoser coupling products were obtained within 70 s residence time. The reaction kinetics was investigated and 15 examples of different building block combinations are given.

## Introduction

**Scheme 1:**
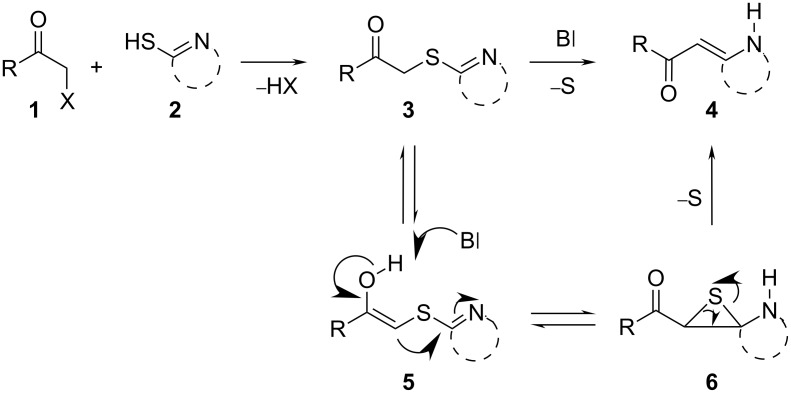
Eschenmoser coupling reaction with secondary S-alkylated thioamide derivatives of type **3**.

The Eschenmoser coupling [1–2] is a reaction method that yields β-enaminocarbonyl derivatives of type **4** by the elimination of sulfur (sulfide contraction) from an episulfide intermediate (Scheme 1). The reaction was described for the first time by Knott in 1955 [3] and became prominent later on when it was applied to the total synthesis of vitamin B12 by Eschenmoser [4]. Since these early days the Eschenmoser coupling has been applied many times as a useful reaction step in a variety of synthesis strategies. Different natural products, such as diplodialid macrolactone- [5], sedamine alkaloid- [6], sparteine- [7], mersicarpine- [8], batzelladine- [9], fuligocandin- [10] and vitamin B12-derivatives [11] were prepared with the aid of sulfide contraction steps. Pharmaceutically important substances, such as methylphenidat [12] or the marine neurotoxin hemibrevetoxin [13], were prepared by utilization of the Eschenmoser coupling reaction. Recently, we explored the Eschenmoser coupling in order to produce Biginelli type dihydropyrimidine (DHPM) derivatives as screening candidates for pharmaceutical and crop science research [14]. Moreover, the Eschenmoser coupling is quite a valuable, metal free, carbon–carbon bond forming reaction.

The necessary starting materials of type **3** can be prepared by S-alkylation of secondary thioamide or thiolactame building blocks of type **2** with α-bromoketones **1** (X = Br). The carbon–carbon coupling occurs between these building blocks as shown in Scheme 1. Due to the ease of accessibility of the building blocks and the usually high-yielding S-alkylation step, the Eschenmoser coupling is an interesting reaction for diversity-oriented combinatorial synthesis [15–17].

The new carbon–carbon bond formation occurs during the construction of the episulfide intermediate **6**, which requires base catalysis (B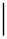). The sulfur extraction from the episulfides **6** or **8** yields the desired β-enaminocarbonyl derivative **4** or **9**. However, the detailed reaction mechanism for the sulfur extraction step has not yet been fully proven and obviously depends on the applied reaction conditions. Experimental observations show that the reaction can take place without the addition of a thiophilic agent or base. This is a strong hint that the sulfide extraction is of an intramolecular pericylic nature, as proposed in Scheme 1. In the presence of a thiophilic agent, the mechanism of extraction via intermediate ionic states also seems plausible. Nevertheless, the Eschenmoser coupling reaction requires the addition of a base and a thiophilic agent in the most cases. Here, triphenylphosphine- or trialkyl-phosphite-derivatives are usually employed to promote the sulfide contraction with valuable yield and selectivity. Eschenmoser himself made use of bi-functional reagents with dual thiophile and basic properties in one molecule [2]. With regards to experimental considerations, the thiophilic reagent must be carefully chosen in order to overcome the difficulties in the separation of the desired product from phosphine sulfide byproducts.

**Scheme 2:**
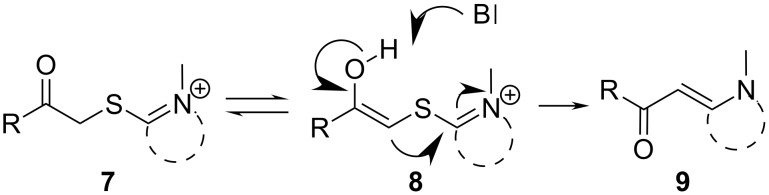
Eschenmoser coupling sequence of S-alkylated ternary thioamides of type **7**.

S-Alkylated ternary thioamides of type **7** usually undergo the episulfide formation and subsequent sulfide contraction smoothly (Scheme 2). This is due to the strong electron accepting nature of the thioiminium intermediate **8**. Unfortunately, the S-alkylation is sometimes difficult in these cases [6]. In the case of secondary thiolactames of type **5**, sulfide contraction takes place as well, but prolonged reaction time and increased reaction temperature are necessary in most cases. In contrast to this, secondary thioamide derivatives of type **5** undergo the S-alkylation with α-bromoketones more readily than ternary thioamides. However, the specific reactivity of both reaction steps obviously depends on the building blocks used and their specific reactivity. For the present work we chose only commercially available α-bromoketone building blocks and secondary thiolactame and thioamide derivatives. To improve the long reaction times we investigated the Eschenmoser coupling under process intensification conditions. Therefore, reaction temperatures far beyond the solvent boiling point were applied under pressurized flow conditions for a minimum residence time. For small scale synthesis flow chemistry is of great advantage to realize these conditions on the laboratory bench safely [18].

## Results and Discussion

**Figure 1:**
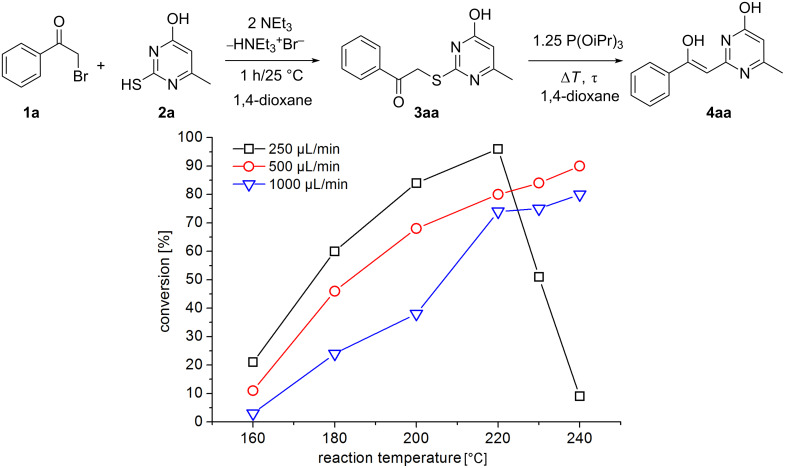
Conversion of **3aa** to **4aa** under different flow conditions.

For the reaction optimization we have focused on a straightforward procedure that finally prevents the isolation of the S-alkylated intermediates **3** and avoids a change of the reaction solvent for the subsequent step. To establish the best conditions we investigated the reaction of 2-bromo-1-phenylethanone (**1a**) with 2-mercapto-6-methyl-pyrimidin-4-ol (**2a**) in detail. Therefore, **2a** was dissolved in different solvents and 2 equiv NEt_3_ were added. After 10 min, the dissolved α-bromoketone **1a** was added dropwise. A concentration of about 0.1 mol/L was achieved in this way. The reaction solutions were sonicated for about 1/2 h. Subsequently, the HNEt_3_Br precipitates were filtered off. The precipitation and filtration was done with care to prevent malfunctions of the feeding pumps. In the case of chloroform as solvent no precipitation took place, but the LC–MS analysis indicated the S-alkylation clearly, with a purity of about 89%. To all filtered solutions 1.25 equiv of triisopropylphosphite (TIP) were added as thiophilic agent before the reaction solutions were fed through the flow chemistry setup. A flow rate of 1000 µL/min (RT: 55 s) was adjusted and the reaction temperature was increased stepwise from 120 to 240 °C. The backpressure regulator was set for all experiments to about 100 bar. The received reaction products were collected and analyzed by LC–MS analysis. In the case of THF as solvent, precipitation took place in the capillary and the backpressure regulator became blocked. In case of chloroform, the reaction solution turned black, and oily polymeric products were formed when the reaction temperature exceeded 150 °C. As a result, only traces of the desired product **4aa** were observed by LC–MS analysis, along with multiple side products. The side products were not investigated in greater detail because of the complexity of the mixture. In the case of ethanol as solvent, the solubility of the starting materials and S-alkylated intermediate **3aa** was poor and the solutes tended to precipitate. However, anhydrous 1,4-dioxane was found to be the best solvent for both reaction steps. Conversions, with anhydrous 1,4-dioxane as solvent, are shown in Figure 1. Only poor conversion was observed up to 160 °C. The maximum conversion of about 98% was observed at 220 °C, with a flow rate of 250 µL/min. At reaction temperatures beyond 220 °C the selectivity of the reaction decreased dramatically and some unidentified decomposition products appeared. Nevertheless, the increase of the flow rate, to about 500 or 750 µL/min at 230 °C, led to comparable good yields and high reaction selectivities. In the case of higher flow rate, of 1000 µL/min and beyond, and the corresponding shorter reaction time, a decreased conversion was observed due to the slow reaction kinetics (Figure 1).

The flow chemistry technique allows the determination of reaction kinetics data in a fast and efficient way, even under pressurized process-identifying conditions. We investigated the kinetics of the sulfide contraction of **3aa** to **4aa** in detail. For this purpose, a 0.1 mol/L reaction solution was fed through the reaction system at different flow rates and reaction temperatures. After each change in the reaction parameters the reaction system was allowed to reach a steady state before a sample was taken. After a change of the flow rate, samples were taken only after a minimum waiting period of about three times the residence time to achieve system equilibration.

In order to compare the reaction performance of the flow reaction with the conventional technique, the same reaction was carried out under batch conditions. The resulting product solutions were analyzed by LC–MS and isolated by the standard precipitation/recrystallization procedure. No conversion was observed in the batch reaction at ambient temperature within 48 h. Even heating up to the boiling point of 1,4-dioxane (101 °C) for about 1 h did not furnish the desired product in any significant amount. However, after refluxing overnight a yield of at least 43% **4aa** was isolated.

**Figure 2:**
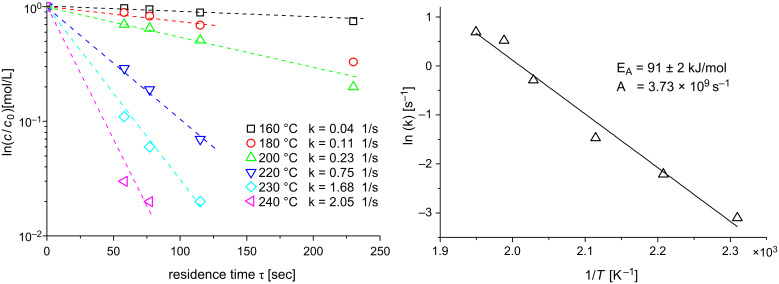
Reaction kinetics analysis. Left: Rate constants with 0.1 M reaction solution. Right: Arrhenius-plot for the determined rate constants.

The investigated Eschenmoser coupling under flow conditions was found to be a first order reaction. Rate constants were calculated using the first order kinetic Equation 1, where *c* [mol/L] is the concentration of the product **4aa** at the reaction temperature *T* [K] after the reaction solution was passed through the heater with the residence time τ [s]. The resulting kinetic plot is shown in Figure 2 (left). The determined rate constants were used to prepare an Arrhenius plot and for calculation of the activation energy from Equation 2. The resulting Arrhenius plot is shown in Figure 2 (right). An activation energy *E*_A_ of about 91 kJ/mol, and a frequency factor *A* of about 3.73 × 10^9^ s^−1^, were found. This activation energy is obviously the reason for the slow reaction kinetics at moderate temperature. Hence, the application of pressurized high temperature conditions should lead to significant reaction intensification.

[1]
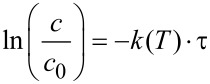


[2]
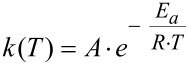


For the analysis of the reaction kinetics the following workflow was necessary: 1) Variation of the reaction parameters, 2) probe sampling, 3) HPLC analysis and 4) reaction kinetics calculations for six different temperatures at five different flow rates. The determination of the complete reaction kinetics data was complete within 6 h. A comparable analysis by a sequential batch technique would take approximately 1 week. Hence, flow chemistry is well suited to efficiently determine the kinetics for reactions under unconventional reaction conditions.

To explore the scope of the sulfide-contraction reaction we varied both building blocks in a systematic manner under the following reaction conditions: 0.1 M reaction solution in anhydrous 1,4-dioxane, 2 equiv NEt_3_, 1.25 equiv TIP at 220 °C and 250–750 µL/min flow rate. First, we varied the thiolactame building blocks **2** and kept the α-bromoketone **1a** constant. In Table 1, the investigated building blocks **2a**–**2i** and the isolated yields are shown. For the product workup, 100% volume of water and 1% 1 M HCl were added to the product solution. If precipitation took place after addition, the residues were filtered. If no precipitation took place, then the reaction solution was extracted with ethyl acetate. The organic phase was separated and dried over MgSO_4_ and the ethyl acetate was subsequently removed by evaporation. The resulting extracts or precipitates were recrystallized from a solvent mixture of hot methanol/dichloromethane. However, the crystallization process was not optimized and there is scope to increase yields further. The desired products **3** were received in synthetically useful yields in almost all cases. The NMR spectra of the resulting products **4** mainly comprise two sets of signals with varying intensities (details can be found in the Supporting Information File 1). This is due to the two different possibilities for the intramolecular H-bond formation caused by the keto–enol tautomerism of **4**. In this scheme, the reaction of 2-mercapto-pyrimidin-4-ol derivatives **2a**, **2b** and **2c** proceeded smoothly with high yields. 6-Aza-2-thiothymine (**2d**) was quantitatively S-alkylated but decomposed during the sulfide-contraction step and subsequent workup. Therefore, we could not isolate the desired product **4ad**. The reaction of dihydropyrimidine substrates **2e** and **2f** proceeded in a facile way, and the desired products **3ae** and **3af** were isolated in good yields. Even the mercapto hydantoin derivative **2g** underwent the two step reaction smoothly and yielded the desired product **4ag** in good quantity. In case of the 1*H*-benzoimidazole-2-thiol (**2h**), the selective S-alkylation failed. The utilized alkylation conditions favor the bis-alkylation, and hence the S-alkylated product was produced in less than 10% yield (HPLC-peak area). LC–MS analysis after the sulfide contraction of this mixture showed that the bis-S-alkylated intermediate was only partially converted. Unfortunately, we could not isolate the product from the complex reaction mixture by the standard precipitation and extraction/crystallization processes. The 2-thiobenzimidazol (**2h**) underwent the S-alkylation as well as the sulfide contraction smoothly and showed good conversion to the desired product **3ah**. However, the applied precipitation technique yielded only a trace amount of the unconverted intermediate **3ah**. *N*-Phenylthioacetamide (**2i**) was additionally chosen as a noncyclic secondary thioamide example. The desired product **4ai** was received in excellent yield.

**Table 1:** Product and yields received for different thio-substrates **2**.

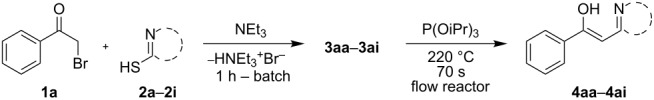				

Structure of **2**		Product **4**		Yield^a^

**2a**	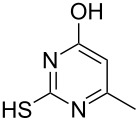	**4aa**	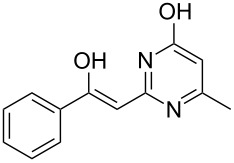	48
**2b**	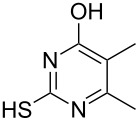	**4ab**	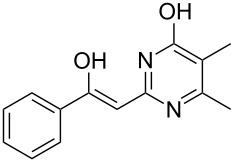	55
**2c**	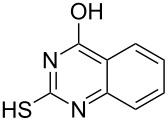	**4ac**	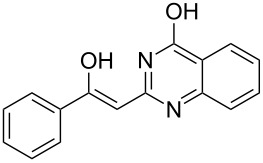	78
**2d**	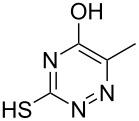	**4ad**	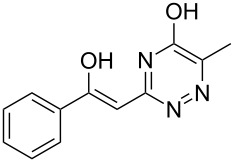	–^b^
**2e**	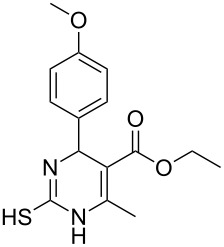	**4ae**	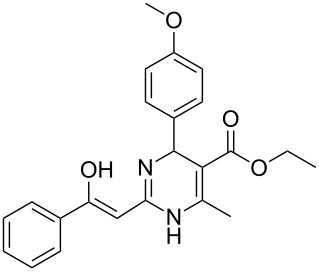	82
**2f**	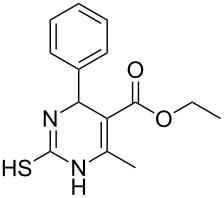	**4af**	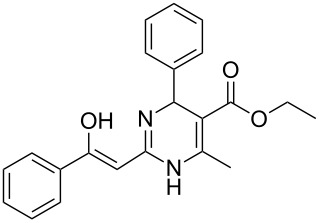	69
**2g**	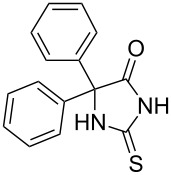	**4ag**	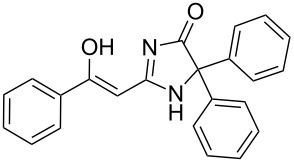	92
**2h**	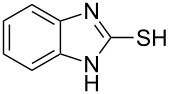	**4ah**	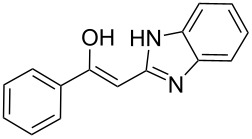	5^c^
**2i**	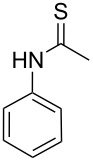	**4ai**	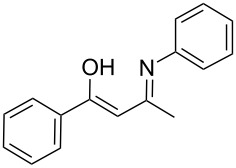	58

^a^Isolated yields; ^b^Decomposition during sulfide contraction; ^c^No product precipitation or extraction of **4ah** was possible; only **3ah** was isolated in smaller amount.

**Scheme 3:**
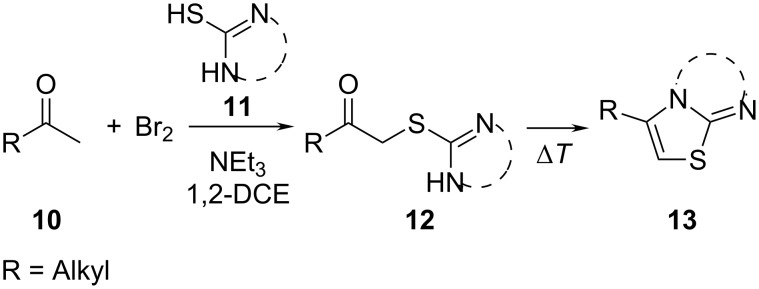
Exclusive formation of thiazol **13** with dihydropyrimidine derivatives **11** take place in the case of aliphatic ketones **10** [19].

Various α-bromoketone building blocks, as shown in Table 2, were investigated in combination with the thiolactame **2a**. We choose only aromatic bromoketones because of their known good reactivity and selectivity for the sulfide contraction [1]. Recently, we investigated the reaction of dihydropyrimidine derivatives of type **11** with different aliphatic ketones **10** under batch conditions. In that case the S-alkylated substrates **12** did not undergo the sulfide contraction but rather the thiazole formation to **13** took place exclusively (Scheme 3) [19]. The thiazol formation by condensation is a well known side reaction of the sulfide contraction. Nevertheless, and in contrast to the batch reaction, no thiazole formation was observed under the investigated flow conditions.

The influence of the bromoketone building blocks **1a**–**1g** on the flow reaction performance was investigated (Table 2). 2-Mercapto-6-methylpyrimidin-4-ol (**2a**) was chosen as the reaction partner for all bromoketones **1a**–**1g**. High yields and reaction selectivities were observed in all cases at the investigated temperature of 220 °C. The influence of the different aromatic bromoketones on the reaction seems to be negligible. The electron accepting nature of the thio-substrate seems to have the dominating effect on the reaction performance. However, all investigated combinations of the building blocks furnished the desired products in good yields within residence times of about 70 s.

**Table 2:** Product and yields for various α-bromoketones **1**.

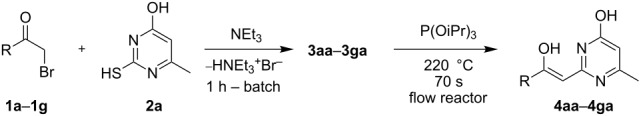				

Structure of **1**		Product **4**		Yield^a^

**1a**	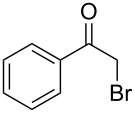	**4aa**	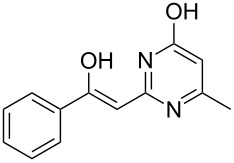	48
**1b**	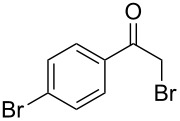	**4ba**	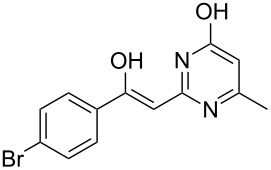	66
**1c**	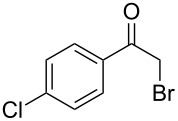	**4ca**	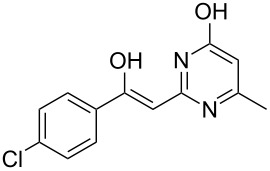	61
**1d**	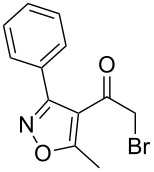	**4da**	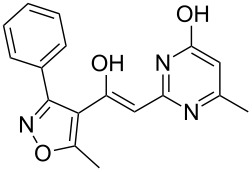	67
**1e**	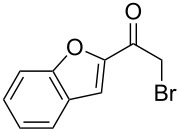	**4ea**	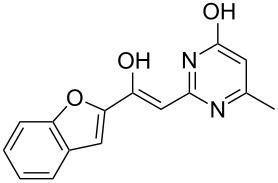	72
**1f**	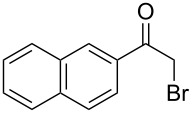	**4fa**	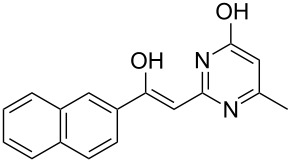	61
**1g**	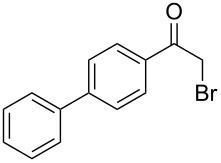	**4ga**	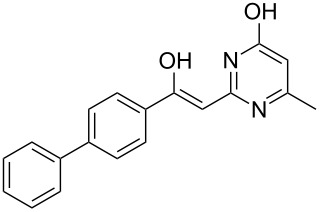	73

^a^Isolated yields.

## Conclusion

We found that Eschenmoser coupling can be performed at a reaction temperature of 220 °C with residence times of about 70 s. 1,4-Dioxane was found to be the most effective solvent. The reaction kinetics was determined in a fast and efficient way for 0.1 M reaction concentration. As a result, the flow chemistry technique enabled the significant intensification of the reaction kinetics, by application of enhanced process conditions on the laboratory bench, in a safe and efficient way. The intensification potential was investigated for demanding secondary thiolactames of type **5**, and exemplary combinations of building blocks, as shown in Table 1 and Table 2, were examined. In almost all investigated cases the desired products were received in synthetically useful yields.

## Experimental

### Flow chemistry system

**Figure 3:**
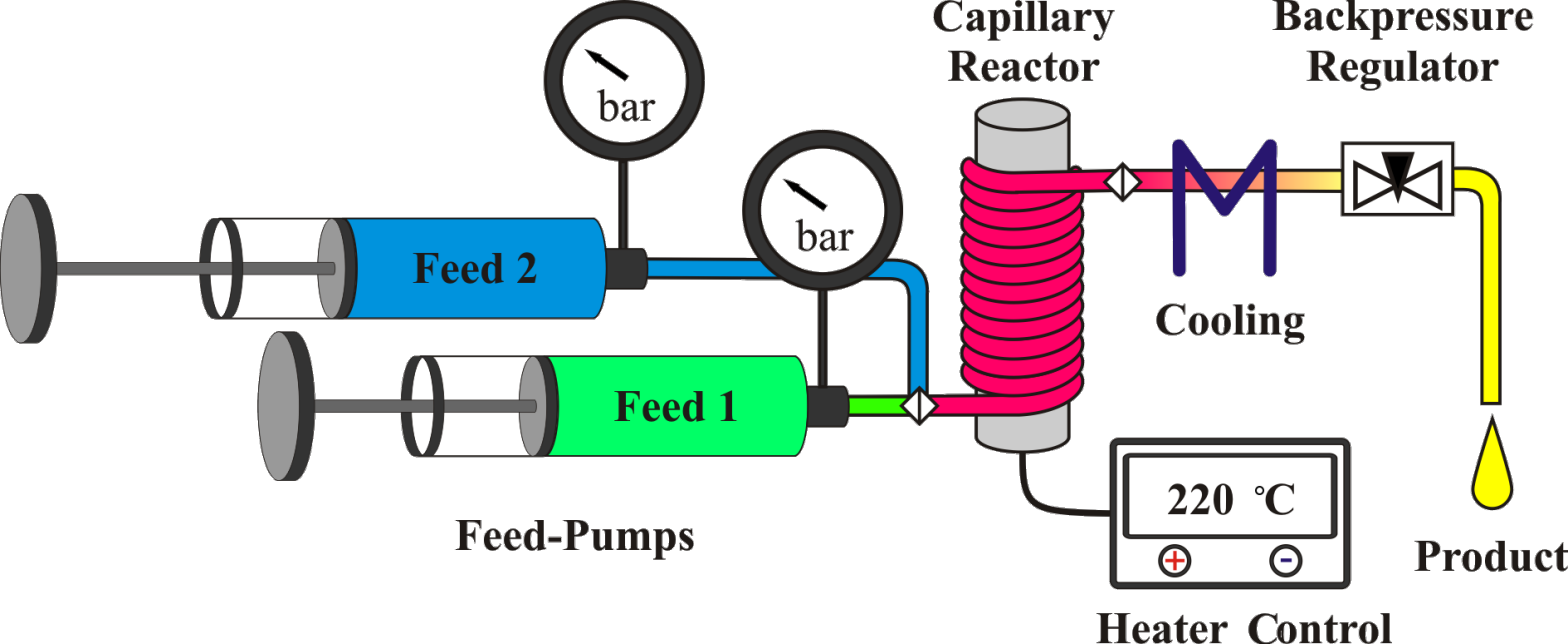
Flow chemistry setup scheme.

**Figure 4:**
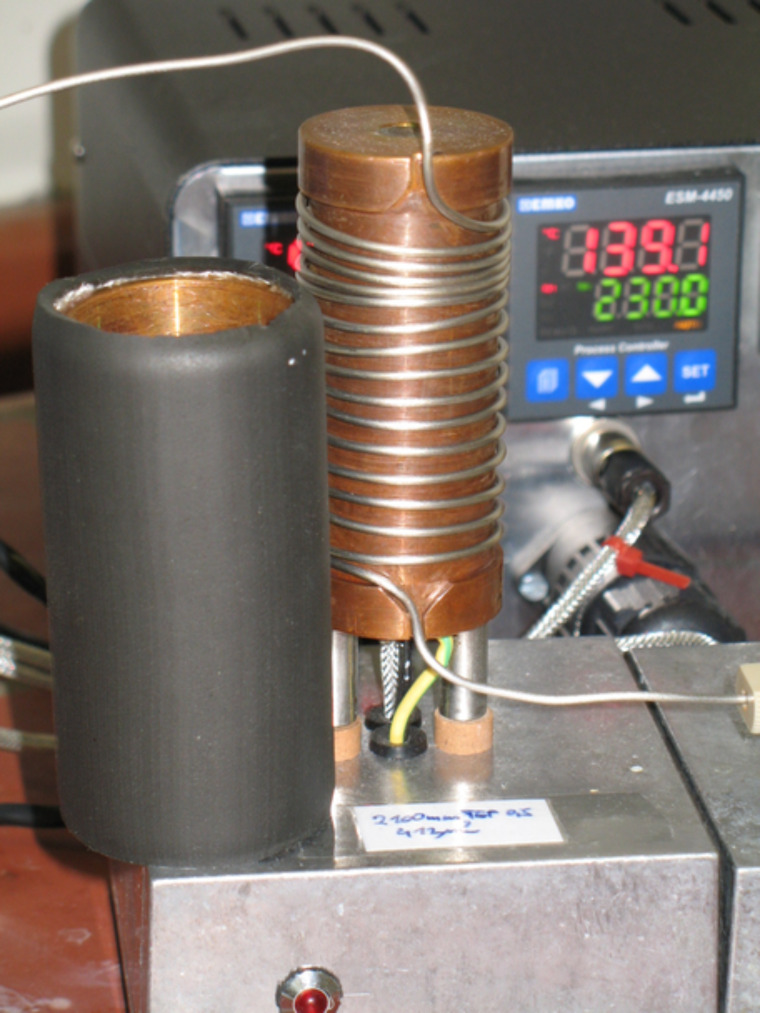
Capillary reactor with jacketed cover removed, and the process controller.

A flow chemistry assembly consisting of two feeding pumps, a capillary reactor, a heat controller and a backpressure regulator was used, as shown in Figure 3. The capillary reactor consists of a 2.1 m long steel capillary with 0.75 mm inner diameter (~930 µL), which was wrapped around a brass cylinder with an integrated heat-rod (hotset, HHP 200 W, 8 × 80 mm) and jacketed by a thermally isolated brass cover (Figure 4). Thermal control was achieved by using a commercial process controller (EMKO process controller ESM-4450) with adapted PT-100 temperature sensor. For pressure control a manual needle valve (micro splitter, Upchurch) was integrated at the capillary outlet. About 20 cm of the capillary between the heating cylinder and the backpressure regulator was tempered by a water chiller to ambient temperature. Feeds were delivered by the help of two double piston HPLC-pumps (Shimadzu LC-9A, double inlet check valves). The first pump was used to feed the reaction solutions and the second pump was used to feed pure solvent for purging and dilution. The maximum operational parameters for the complete flow chemistry setup were proven up to 250 °C and 200 bar. Residence times (τ) between 40 min and 60 s were achieved by adjusting the flow rate from 20 to 2000 µL/min.

## Supporting Information

File 1 General procedures, analytical and spectral data.

## References

[1] Shiosaki K, Trost B M (1991). The Eschenmoser Coupling Reaction. Comprehensive Organic Synthesis.

[2] Roth M, Dubs P, Götschi E, Eschenmoser A (1971). Helv Chim Acta.

[3] Knott E B (1955). J Chem Soc.

[4] Eschenmoser A, Wintner C E (1977). Science.

[5] Ireland R E, Brown F R (1980). J Org Chem.

[6] Neto B A D, Lapis A A M, Bernd A B, Russowsky D (2009). Tetrahedron.

[7] Wlodarczak J, Wysocka W, Katrusiak A (2010). J Mol Struct.

[8] Nakajima R, Ogino T, Yokoshima S, Fukuyama T (2010). J Am Chem Soc.

[9] Elliott M C, Long M S (2004). Org Biomol Chem.

[10] Pettersson B, Hasimbegovic V, Bergman J (2011). J Org Chem.

[11] Mulzer J, List B, Bats J W (1997). J Am Chem Soc.

[12] Russowsky D, da Silveira Neto B A (2003). Tetrahedron Lett.

[13] Nicolaou K C, Reddy K R, Skokotas G, Sato F, Xiao X Y, Hwang C K (1993). J Am Chem Soc.

[14] Singh S, Schober A, Gebinoga M, Groß G A (2009). Tetrahedron Lett.

[15] Groß G A, Wurziger H, Schlingloff G, Schober A (2006). QSAR & Comb Sci (Special Issue: Array Synthesis).

[16] Groß G A, Mayer G, Albert J, Riester D, Osterodt J, Wurziger H, Schober A (2006). Angew Chem, Int Ed.

[17] Gebinoga M, Groß G A, Albrecht A, Lübeck T, Henkel T, Hoffmann P, Klemm U, Schlingloff G, Frank T, Schober A (2006). QSAR & Comb Sci (Special Issue: Array Synthesis).

[18] Wegner J, Ceylan S, Kirschning A (2011). Chem Commun.

[19] Singh S, Schober A, Gebinoga M, Groß G A (2011). Tetrahedron Lett.

