# Supplementary material for: The Eschenmoser coupling reaction under continuous-flow conditions
Source: Beilstein J Org Chem. 2011 Aug 25;7:1164–72. doi: 10.3762/bjoc.7.135 (PMC3170046; doi:10.3762/bjoc.7.135)

# Supporting Information

for

## The Eschenmoser coupling reaction under continuous-flow conditions

Sukhdeep Singh, J. Michael Köhler, Andreas Schober and G. Alexander Groß*

Address: Institute for Chemistry and Biotechnology, Technische Universität Ilmenau, Weimarerstr. 32, D-98693-Ilmenau

Email: Sukhdeep Singh - Sukhdeep.Singh@tu-ilmenau.de, J. Michael Köhler - michael.koehler@tu-ilmenau.de, Andreas Schober - Andreas.Schober@tu-ilmenau.de, G. Alexander Groß* - alexander.gross@tu-ilmenau.de

* Corresponding author

## General procedures, analytical and spectral data

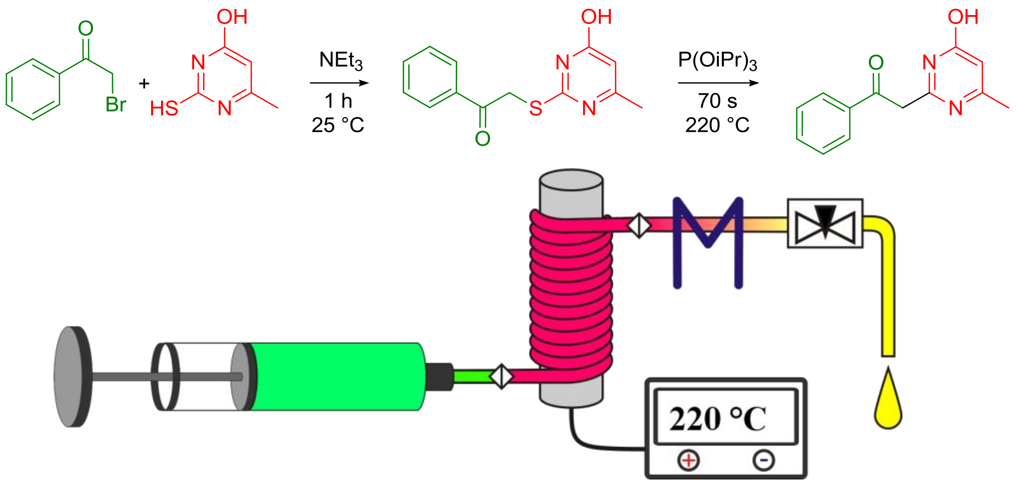

## Materials and methods

$^1H/^{13}C$ NMR (300/75 or 200/50 MHz; DMSO, $CDCl_3$) spectra were recorded, with commercial deuterated solvents, on a multinuclear spectrometer Bruker Avance 300 MHz or 200 MHz. Data analysis was performed by using MestReNova v5.3.2-4936 software. Data are reported as follows: Chemical shifts in ppm (multiplicity [singlet (s), doublet (d), double doublet (dd) triplet (t), quartet (q), AB quartet (ABq), broad (br), and multiplet (m)], coupling constant [Hz], integration). High resolution mass spectra (HRMS) were recorded on a Bruker Daltonics MicroTOF Focus spectrometer using Na- and Li-formate solutions for internal calibration. IR spectra were recorded on a Jasco FTIR 6300 spectrophotometer. A Jasco 2000 HPLC equipped with a DAD-detector was coupled to the Bruker Daltonics MicroTOF focus mass spectrometer to investigate the reactions by LC-MS analysis. Merck Chromolith 100 × 2 mm PR18 column was used with water + 0.1% TFA (A)/acetonitrile + 0.1 % TFA (B) eluents at 750 μL/min. Gradient method: 1 min 15% B; 2 min 35% B; 2.5 min 35% B; 4 min 95% B. All reaction solvents were dried overnight with 4Å molecular sieves before use. All commercially available building blocks and reagents were used as received without any further purification.

## Representative procedure

### 1.) S-Alkylation in batch:

2-Mercapto-6-methylpyrimidin-4-ol (**2a**) (1136 mg, 8 mmol, 1 equiv) was dissolved in 50 mL anhydrous 1,4-dioxane. Triethylamine (2288 µL, 16.4 mmol, 2.1 equiv) was added dropwise under magnetic stirring. 2-Bromoacetophenone (**1a**, 1592 mg, 8 mmol, 1 equiv) was dissolved in 30 mL anhydrous 1,4-dioxane and added to the first solution. The mixture was placed in an ultrasonic bath for about 1 h at about 60 °C. After cooling to ambient temperature, the precipitates were carefully filtered.

### 2.) Sulfide contraction in flow

Triisopropylphosphite (TIP, 1868 µL, 8.2 mmol, 1.2 equiv) was added immediately before the reaction solution was passed through the flow reactor. The

reaction mixture was fed at a rate of 500 µL/min through the capillary reactor at 220 °C. The capillary was prefilled at high temperature with pure 1,4-dioxane to prevent boiling and precipitation during the ramp-up process. A system pressure of at least 100 bar was adjusted by the backpressure regulator.

For workup, 50 mL of the reaction solution was collected at the capillary outlet and 50 mL water as well as 0.5 mL 1 N HCl were added. After vigorous shaking, the solution was kept cold for about 30 min to obtain precipitates, which were filtered and washed with acetone. After drying under vacuum, 880 mg (3.8 mmol, 48%) 2-(2-hydroxy-2-phenylvinyl)-6-methylpyrimidin-4-ol (**4aa**) was obtained as off-white solid. The LC-MS and HR-MS analysis of the obtained precipitates showned only a single pure compound. However, in the $^{1}$H NMR analyses a duplicate set of signals was found indicating a mixture of two isomers. Recrystallization from hot methanol and DCM helped in enriching one isomer over the other.

## Summarized analytical data:

**2-(2-Hydroxy-2-phenylvinyl)-6-methylpyrimidin-4-ol (4aa)**

Isolated yield 48%; off-white Solid; $\nu_{max}$ (neat) 1653, 1619, 1525, 1353, 1217 cm$^{-1}$; $^{1}$H NMR (300 MHz, DMSO): δ 2.15, 2.23 (s, 3H), 5.56, 5.65 (s, 1H, exch. with $D_2O$), 5.73, 5.81 (s, 1H, exch. with $D_2O$), 7.49 (m, 3H), 7.99 (m, 2H), 11.48, 11.67 (br, 1H, exch. with $D_2O$), 13.67 (br, 1H, exch. with $D_2O$) ; $^{13}$C NMR (75 MHz, DMSO): δ 18.5, 19.3, 66.3, 77.4, 79.2, 101.5, 103.5, 126.1, 126.3, 128.1, 128.4, 128.8, 130.8, 133.6, 138.5, 139.5, 153.0, 154.4, 155.5, 159.8, 160.8, 182.8, 185.6; HRMS (EI, Li-formate calibrant) calcd. for $C_{13}H_{13}N_2O_2$ [MH]$^{+}$: 229.09715; found [MH]$^{+}$ 229.09716.

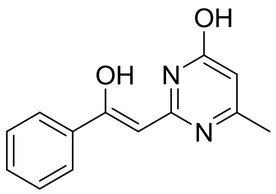


**2-(2-Hydroxy-2-phenylvinyl)-5,6-dimethylpyrimidin-4-ol (4ab)**

Isolated yield 55%; yellow solid; $\nu_{max}$ (neat) 1620, 1448, 1310 cm$^{-1}$; $^{1}$H NMR (300 MHz, DMSO): δ 1.84 (s, 3H), 2.22 (s, 3H), 5.75 (s. 1H), 7.48 (m, 3H), 7.76 (m, 2H), 11.39, 11.88 (br, 1H, exch. with $D_2O$), 13.95, 14.59 (br, 1H, exch. with $D_2O$); $^{13}$C NMR (75 MHz, DMSO): δ 9.9, 16.5, 17.8, 66.3, 76.6, 78.7, 110.5, 126.0, 128.4, 130.6, 138.4, 147.7, 154.2; HRMS (EI, Li-formate calibrant) calcd. for $C_{14}H_{15}N_2O_2$ [MH]$^{+}$: 243.11280; found [MH]$^{+}$ 243.11294.

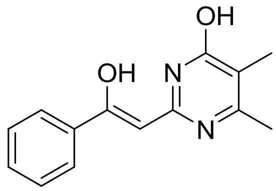


**2-(2-Hydroxy-2-phenylvinyl)quinazolin-4-ol (4ac)**

Isolated yield 78%; yellow solid; $\nu_{max}$ (neat) 1675, 1633, 1539, 1473, 1295, 1142 cm$^{-1}$; $^{1}$H NMR (300 MHz, DMSO): δ 5.79, 5.91 (s, 1H) 7.29–8.00 (m, 9H), 11.86, 12.00 (s, 1H, exch. with $D_2O$), 14.15, 14.62 (s, 1H, exch. with $D_2O$); $^{13}$C NMR (75 MHz, DMSO): δ 66.3, 77.8, 79.8, 116.3, 117.1, 118.3, 126.1, 126.4, 128.5, 130.9, 135.3, 138.4, 140.3, 153.1, 153.6, 160.0, 182.5; HRMS (EI, Li-formate calibrant) calcd. for $C_{16}H_{13}N_2O_2$ [MH]$^{+}$: 265.09715; found [MH]$^{+}$ 265.09713.

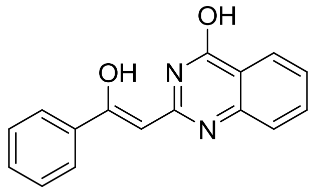

**5-Ethoxycarbonyl-6-methyl-4-(4-methoxyphenyl)-2-(2-hydroxy-2-phenylvinyl)-1,4-dihydropyrimidine (4ae)**

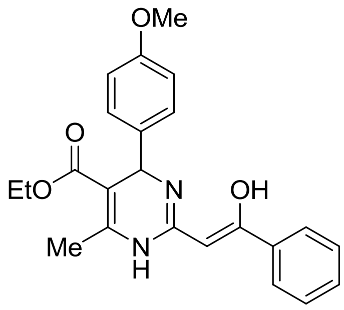


Isolated yield 82%; creamy solid; $\nu_{max}$ (neat) 2931, 1685, 1628, 1574, 1531, 1473 cm$^{-1}$; $^{1}$H NMR (200 MHz, CDCl$_3$): δ 1.18 (t, 3H, *J* = 8.0 Hz), 2.45, 2.38 (s, 3H), 3.76, 3.73 (s, 3H), 4.08 (m, 3H), 5.24 (s, 1H), 5.29, 5.41 (s, 1H), 5.42 (br, 1H, exch. with D$_2$O), 6.76–7.65 (m, 9H, ArH), 12.83, 11.39 (br, 1H, exch. with D$_2$O); $^{13}$C NMR (50 MHz, CDCl$_3$): δ 14.2, 19.5, 52.8, 55.3, 60.3, 78.7, 103.5, 114.2, 127.7, 128.3, 131.5, 135.9, 139.3, 143.3, 154.7, 159.4, 165.5, 185.5; HRMS (EI, Li-formate calibrant) calcd. for $C_{23}H_{25}N_2O_4$ [MH]$^+$: 393.1809; found [MH]$^+$ 393.1810.

**5-Ethoxycarbonyl-6-methyl-4-phenyl-2-(2-hydroxy-2-phenylvinyl)-1,4-dihydropyrimidine (4af)**

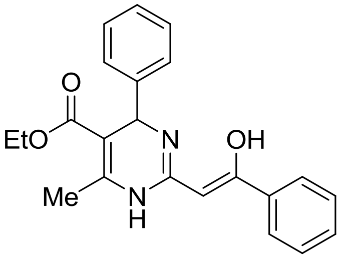


Isolated yield 69%; dark yellow viscous liquid; $\nu_{max}$ (neat) 2927, 1629, 1583, 1529, 1478 cm$^{-1}$; $^{1}$H NMR (200 MHz, CDCl$_3$): δ 1.18, 1.21 (t, 3H, *J* = 6.2 and 6.0 Hz), 2.48, 2.40 (s, 3H), 4.09 (q, 2H, *J* = 6.0 Hz), 5.33, 5.29 (1H, exch. with D$_2$O), 5.36, 5.49 (s, 1H), 7.31–7.80 (m, 10H), 12.94, 11.48 (br, 1H, exch. with D$_2$O); $^{13}$C NMR (50 MHz, CDCl$_3$): δ 14.2, 19.5, 53.4, 59.9, 78.3, 102.9, 110.9, 126.4, 128.2, 128.9, 130.8, 140.1, 154.5, 165.4; HRMS (EI, Li-formate calibrant) calcd. for $C_{22}H_{23}N_2O_3$ [MH]$^+$: 363.1703; found [MH]$^+$ 363.1703.

**2-(2-Hydroxy-2-phenylvinyl)-5,5-diphenyl-1,5-dihydroimidazol-4-one (4ag)**

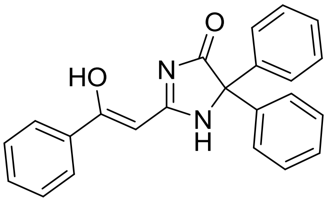


Isolated yield 92%; white solid; $\nu_{max}$ (neat) 1728, 1634, 1517, 1479, 1430, 1337, 1295 cm$^{-1}$; $^{1}$H NMR (300 MHz, DMSO): δ 5.71, 5.76 (s, 1H) 7.39–7.87 (m, 15H), 10.57, 10.01 (br, 1H, exch. with D$_2$O), 11.78 (br, 1H, exch. with D$_2$O); $^{13}$C NMR (75 MHz, DMSO): δ 23.5, 66.3, 68.4, 70.2, 75.6, 75.9, 126.5, 126.6, 126.8, 128.2, 128.3, 128.4, 128.5, 128.7, 131.0, 139.3, 139.4, 157.3,

158.0, 173.8, 186.7; HRMS (EI, Li-formate calibrant) calcd. for $C_{23}H_{19}N_2O_2$ $[MH]^+$: 355.14410; found $[MH]^+$ 355.14390.

**2-(1*H*-Benzimidazol-2-ylsulfanyl)-1-phenylethanone (3ah)**

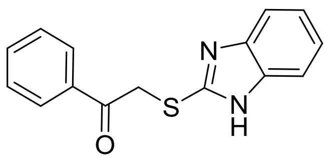


Isolated yield <5%; yellow solid; $^1$H NMR (300 MHz, DMSO/$CDCl_3$): δ 5.20 (s, 2H), 7.23–8.07 (m, 9H); $^{13}$C NMR (75 MHz, DMSO/$CDCl_3$): δ 91.61, 122.7, 128.4, 128.7, 133.8, 135.1, 191.6; HRMS (EI, Li-formate calibrant) calcd. for $C_{15}H_{13}N_2OS$ $[MH]^+$: 269.07431; found $[MH]^+$ 269.07461.

**1-Phenyl-3-phenylimino-but-1-en-1-ol (4ai)**

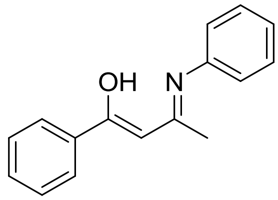


Isolated yield 58%; yellow solid; $\nu_{max}$ (neat) 1587, 1544, 1519, 1317, 1193 cm$^{-1}$; $^1$H NMR (300 MHz, DMSO): δ 6.10 (s, 1H), 7.33–7.96 (m, 10H), 13.17 (s, 1H, exch. with $D_2O$); $^{13}$C NMR (75 MHz, DMSO): δ 19.9, 93.8, 124.0, 125.5, 126.8, 128.3, 129.3, 131.0, 138.2, 139.3, 162.4, 187.0; HRMS (EI, Li-formate calibrant) calcd. for $C_{16}H_{16}NO$ $[MH]^+$: 238.12264; found $[MH]^+$ 238.12252.

**2-[2-(4-Bromophenyl)-2-hydroxyvinyl]-6-methylpyrimidin-4-ol (4ba)**

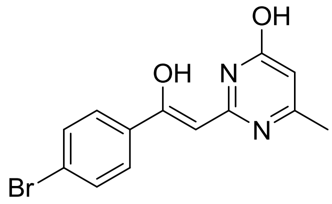


Isolated yield 66%; off-white solid; $\nu_{max}$ (neat) 1621, 1576, 1392, 1349, 1214, 1046, 763 cm$^{-1}$; $^1$H NMR (300 MHz, DMSO): δ 2.16, 2.24 (s, 3H), 5.69, 5.61 (s, 1H), 5.76, 5.68 (s, 1H), 7.69 (m, 4H), 11.74 (br, 1H, exch. with $D_2O$), 14.28, 13.64 (s, 1H, exch. with $D_2O$); $^{13}$C NMR (75 MHz, DMSO): δ 18.5, 19.1, 67.7, 77.3, 78.5, 101.8, 103.5, 124.5, 131.5, 137.8, 138.5, 152.4, 152.8, 154.6, 155.4, 159.6, 160.7, 184.0; HRMS (EI, Li-formate calibrant) calcd. for $C_{13}H_{12}BrN_2O_2Na$ $[MH]^+$: 328.98961; found $[MH]^+$ 328.98984.

**2-[2-(4-Chlorophenyl)-2-hydroxyvinyl]-6-methylpyrimidin-4-ol (4ca)**

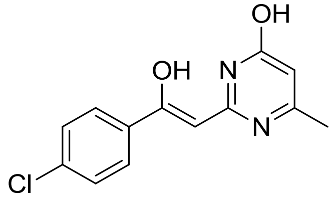


Isolated yield 61%; yellow crystalline solid; $\nu_{max}$ (neat) 1621, 1579, 1351, 1215, 1044, 762 cm$^{-1}$; $^{1}$H NMR (300 MHz, DMSO): δ 2.20 (s, 3H), 5.65 (s, 1H), 5.73 (s, 1H), 7.55 (d, 2H, *J* = 9.0 Hz), 7.78 (d, 2H, *J* = 9.0 Hz), 11.79 (br, 1H, exch. with $D_2O$), 14.26, 13.63 (s, 1H, exch. with $D_2O$); $^{13}$C NMR (75 MHz, DMSO): δ 18.8, 99.5, 128.1, 128.5, 135.6, 152.6, 154.9; HRMS (EI, Li-formate calibrant) calcd. for $C_{13}H_{12}ClN_2O_2$ [MH]$^+$: 263.05818; found [MH]$^+$ 263.05811.

**2-[2-Hydroxy-2-(5-methyl-3-phenylisoxazol-4-yl)vinyl]-6-methylpyrimidin-4-ol (4da)**

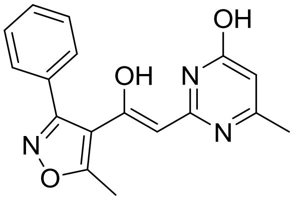


Isolated yield 67%; off-white solid; $\nu_{max}$ (neat) 1667, 1621, 1541, 1418, 1248, 1158 cm$^{-1}$; $^{1}$H NMR (300 MHz, DMSO): δ 2.10, 2.21 (s, 3H), 2.60 (s, 3H), 5.02, 5.10 (s, 1H), 5.59, 5.64 (s, 1H), 7.45–7.63 (m, 5H), 11.49, 11.68 (br, 1H, exch. with $D_2O$), 13.43, 13.81 (br, 1H, exch. with $D_2O$); $^{13}$C NMR (75 MHz, DMSO): δ 12.3, 18.4, 19.0, 82.2, 83.1, 101.9, 103.4, 116.6, 117.1, 128.4, 128.5, 128.6, 129.7, 152.0, 152.8, 154.0, 154.6, 159.5, 160.4, 160.6, 170.0, 179.0, 180.4; HRMS (EI, Li-formate calibrant) calcd. for $C_{17}H_{15}N_3O_3Na$ [MH]$^+$: 332.10056; found [MH]$^+$ 332.10078.

**2-[(2-Benzofuran-2-yl)-2-hydroxyvinyl]-6-methylpyrimidin-4-ol (4ea)**

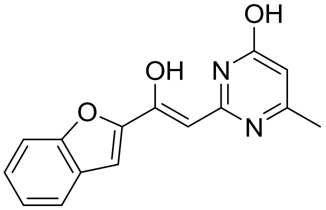


Isolated yield 72%; off-white solid; $\nu_{max}$ (neat) 1632, 1537, 1389, 1360, 1344, 1227, 1049 cm$^{-1}$; $^{1}$H NMR (300 MHz, DMSO): δ 2.17, 2.25 (s, 3H), 5.65, 5.71 (s, 1H), 5.85, 5.88 (s, 1H), 7.30–7.71 (m, 5H), 11.83 (br, 1H, exch. with $D_2O$), 13.39, 14.07 (br, 1H, exch. with $D_2O$); $^{13}$C NMR (75 MHz, DMSO): δ 18.5, 19.0, 78.1, 79.1, 102.1, 103.1,

107.9, 111.7, 122.5, 123.5, 126.5, 127.5, 152.2, 152.8, 154.3, 154.6, 155.3, 159.4, 160.6, 174.2, 175.5; HRMS (EI, Li-formate calibrant) calcd. for $C_{15}H_{13}N_2O_3$ $[MH]^+$: 269.09207; found $[MH]^+$ 269.09200.

**2-(2-Hydroxy-2-(naphthalen-2-yl)vinyl)-6-methylpyrimidin-4-ol (4fa)**

Isolated yield 61%; off-white solid; $\nu_{max}$ (neat) 1653, 1557, 1428, 1295 $cm^{-1}$; $^1H$ NMR (300 MHz, DMSO): δ 2.17, 2.26 (s, 3H), 5.61, 5.70 (s, 1H), 5.89, 5.96 (s, 1H), 7.59–8.38 (m, 7H), 11.62, 11.81 (br, 1H, exch. with $D_2O$), 13.75, 14.52 (br, 1H, exch. with $D_2O$); $^{13}C$ NMR (75 MHz, DMSO): δ 18.5, 19.3, 66.3, 77.8, 79.4, 101.6, 103.5, 123.2, 123.5, 127.5, 132.3, 134.0, 135.9, 136.8, 152.8, 152.9, 154.4, 155.4, 159.7, 160.8, 182.7, 185.2; HRMS (EI, Li-formate calibrant) calcd. for $C_{17}H_{14}N_2O_2$ $[MH]^+$: 279.11280; found $[MH]^+$ 279.11291.

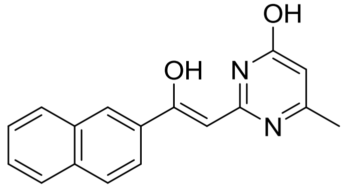


**2-(2-(1,1'-Biphenyl-4-yl)2-hydroxyvinyl)-6-methylpyrimidin-4-ol (4ga)**

Isolated yield 73%; yellow solid; $\nu_{max}$ (neat) 1653, 1567, 1421, 1303 $cm^{-1}$; $^1H$ NMR (300 MHz, DMSO): δ 2.17, 2.25 (s, 3H), 5.60, 5.68 (s, 1H), 5.78, 5.86 (s, 1H), 7.41–7.90 (m, 9H), 11.56, 11.79 (br, 1H, exch. with $D_2O$), 13.74, 14.44 (br, 1H, exch. with $D_2O$); $^{13}C$ NMR (75 MHz, DMSO): δ 18.5,19.3, 66.3, 77.4, 78.9, 101.5, 103.4, 126.7, 126.8, 129.0, 137.4, 138.3, 139.2, 142.4, 152.7, 152.8, 154.4, 155.4, 159.7, 160.8, 182.4, 184.9; HRMS (EI, Li-formate calibrant) calcd. for $C_{19}H_{17}N_2O_2$ $[MH]^+$: 305.12845; found $[MH]^+$ 305.12858.

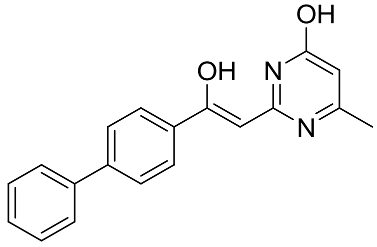

**Spectral data:** $^{1}H/^{13}C$ NMR (300/75 MHz or 200/50 MHz; DMSO/$CDCl_3$) spectra were recorded with commercial deuterated solvents on a multinuclear spectrometer Bruker Avance 300/200 MHz.

**2-(2-Hydroxy-2-phenylvinyl)-6-methylpyrimidin-4-ol (4aa, first crop)**

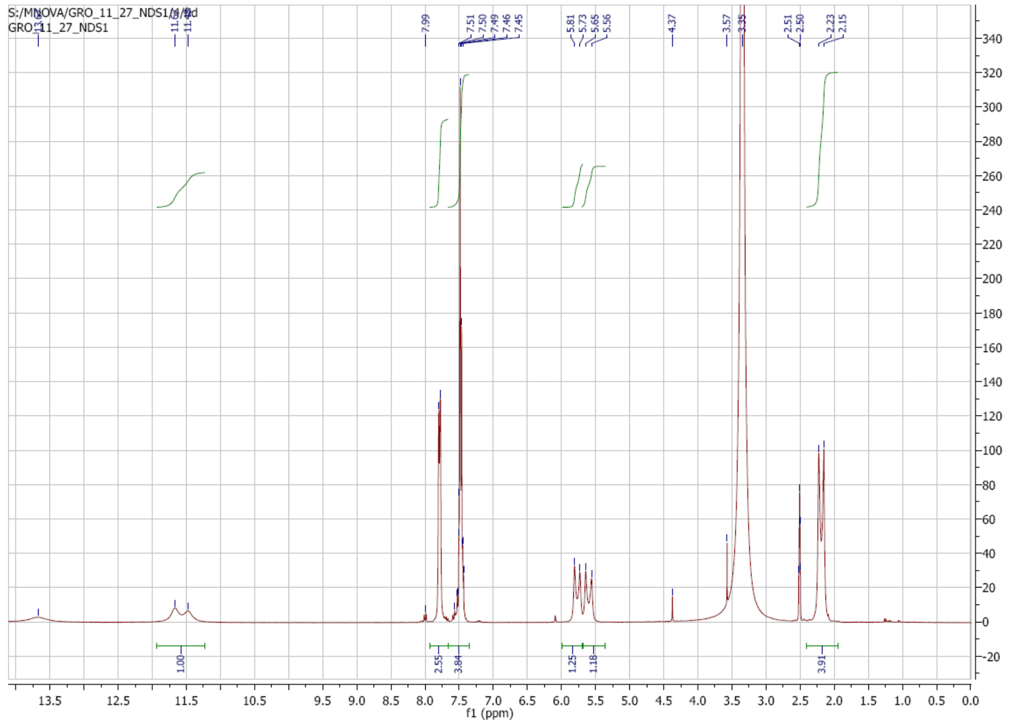


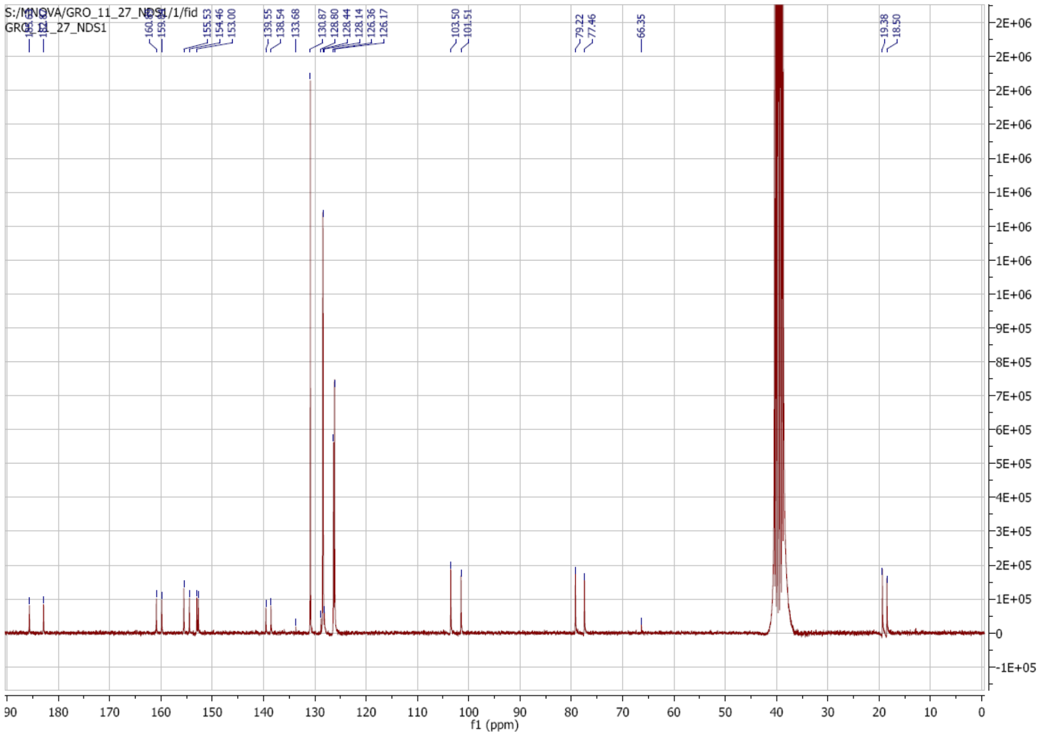

**2-(2-Hydroxy-2-phenylvinyl)-6-methylpyrimidin-4-ol (4aa, recrystallized from methanol/DCM)**

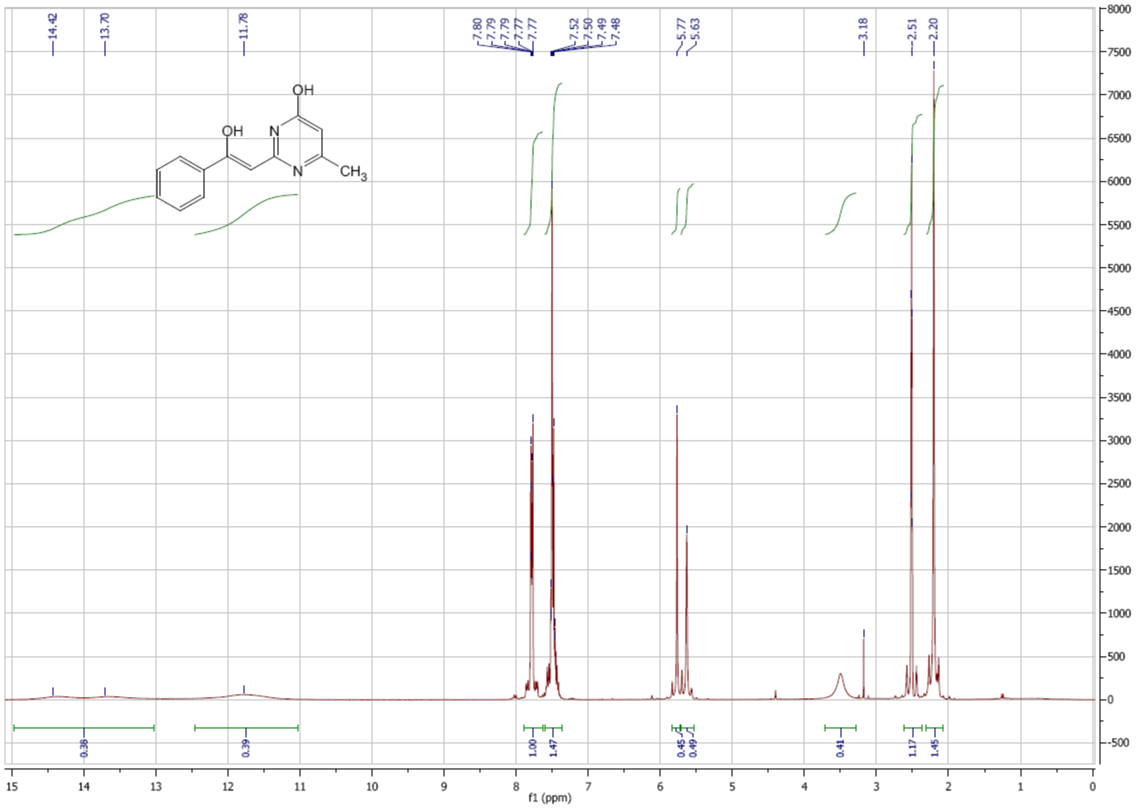

## 2-(2-Hydroxy-2-phenylvinyl)-5,6-dimethylpyrimidin-4-ol (4ab)

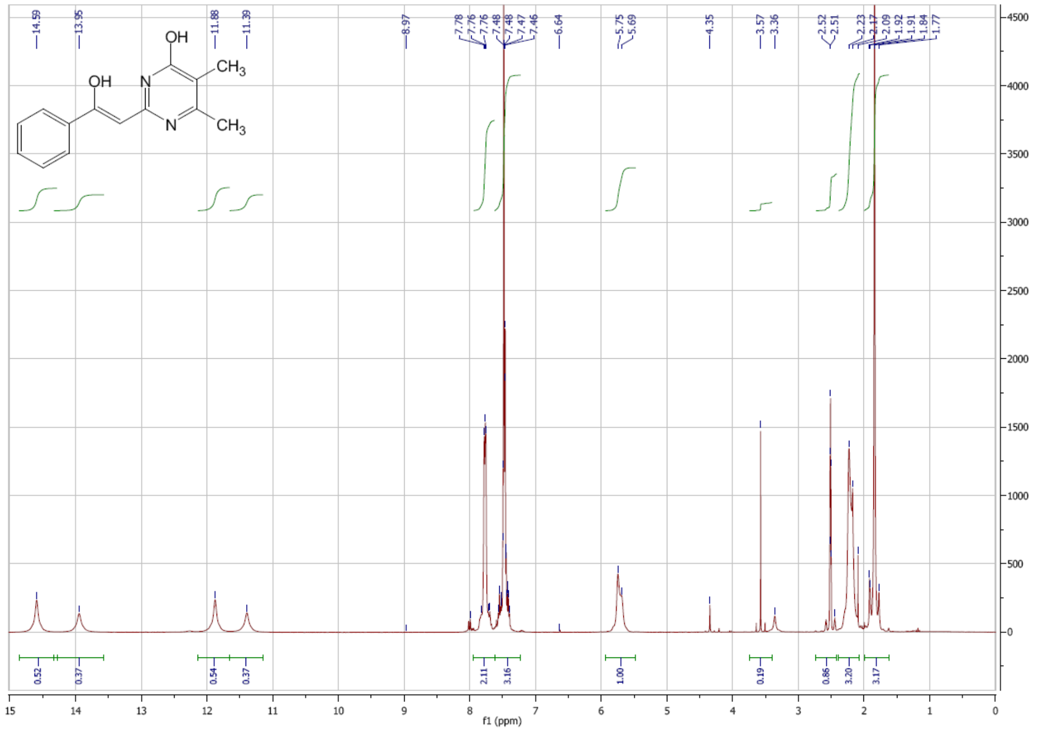

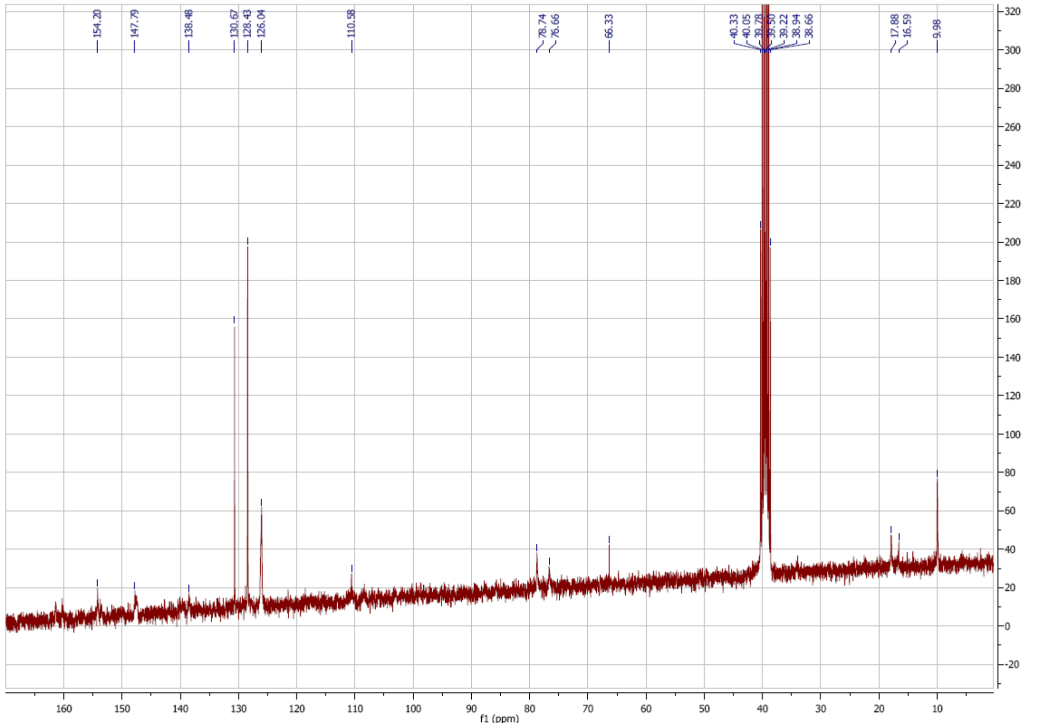

## 2-(2-Hydroxy-2-phenylvinyl)quinazolin-4-ol (4ac)

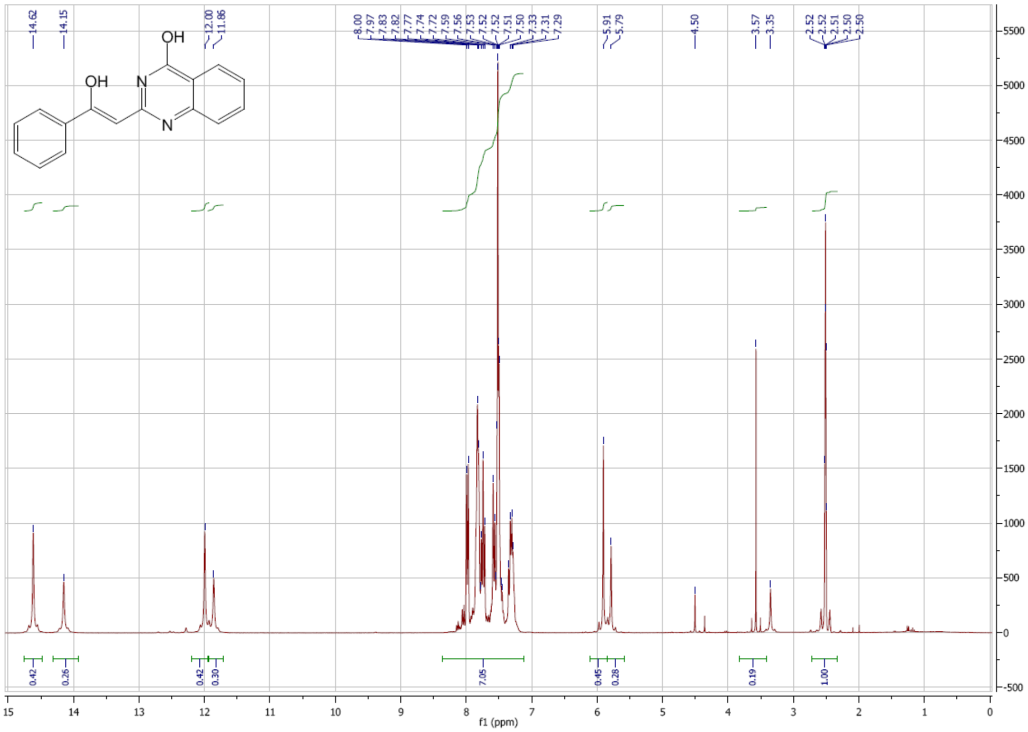


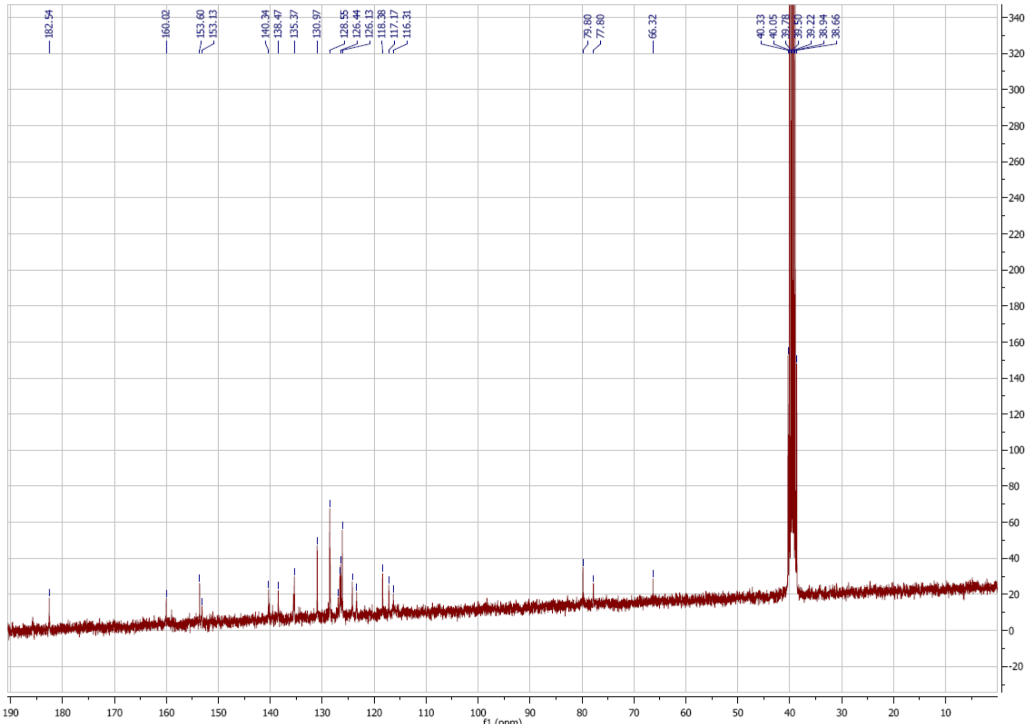

**5-Ethoxycarbonyl-6-methyl-4-(4-methoxyphenyl)-2-(2-hydroxy-2-phenylvinyl)-1,4-dihydropyrimidine (4ae)**

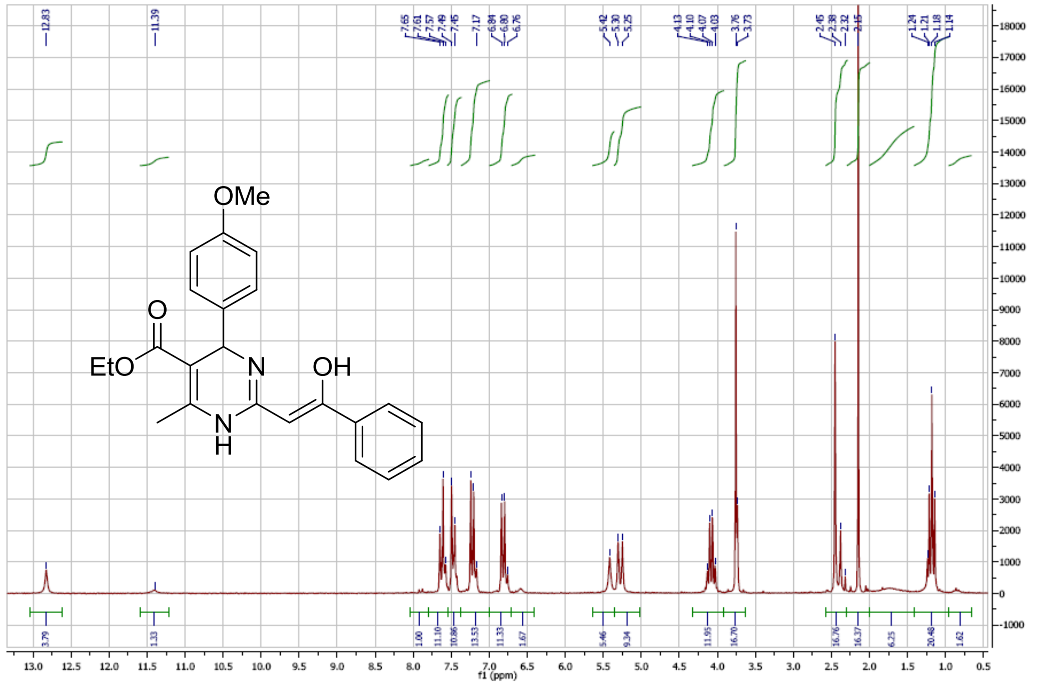

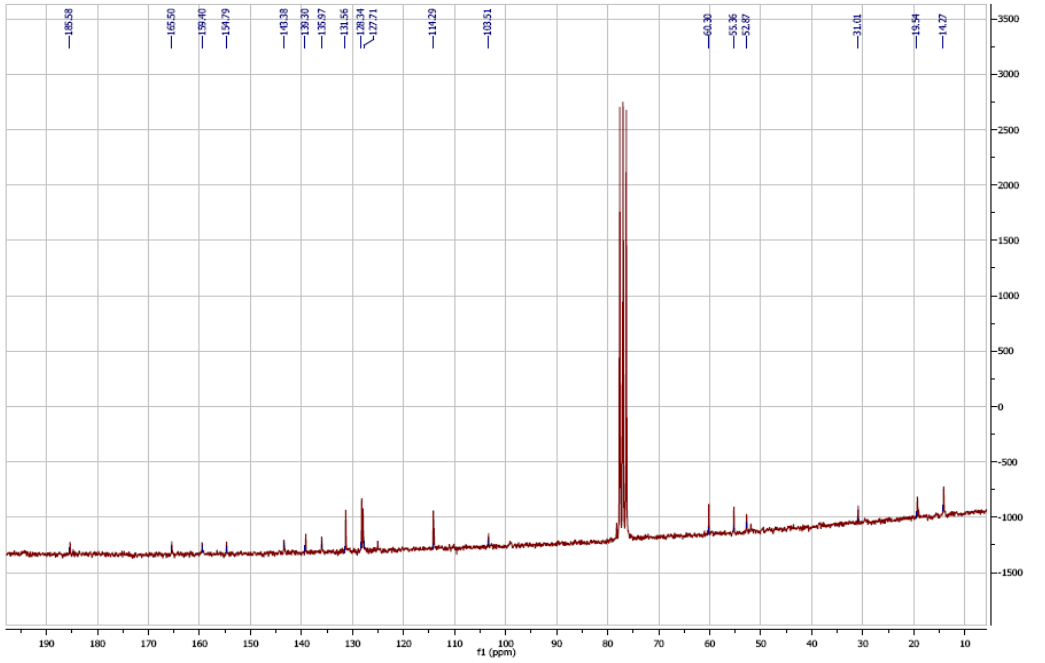

**5-Ethoxycarbonyl-6-methyl-4-phenyl-2-(2-hydroxy-2-phenylvinyl)-1,4-dihydropyrimidine (4af)**

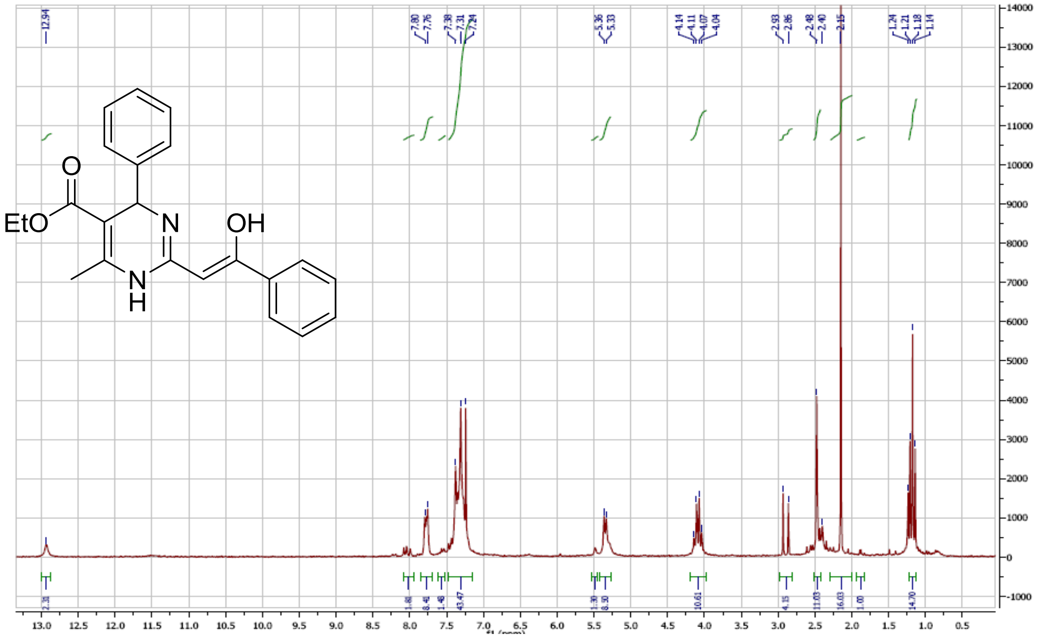


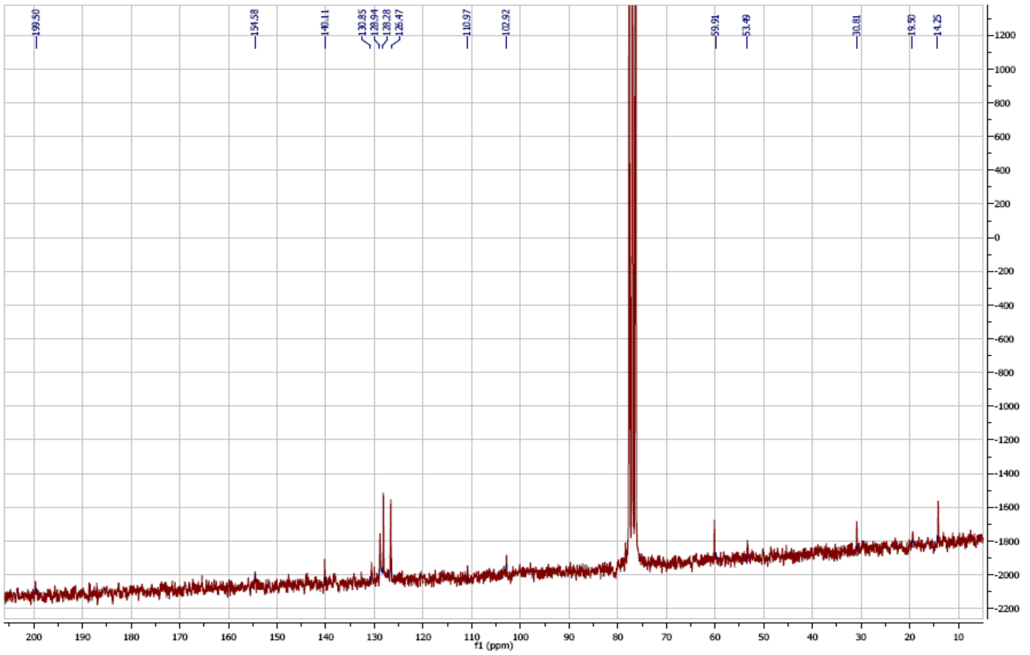

**2-(2-Hydroxy-2-phenylvinyl)-5,5-diphenyl-1,5-dihydroimidazol-4-one (4ag)**

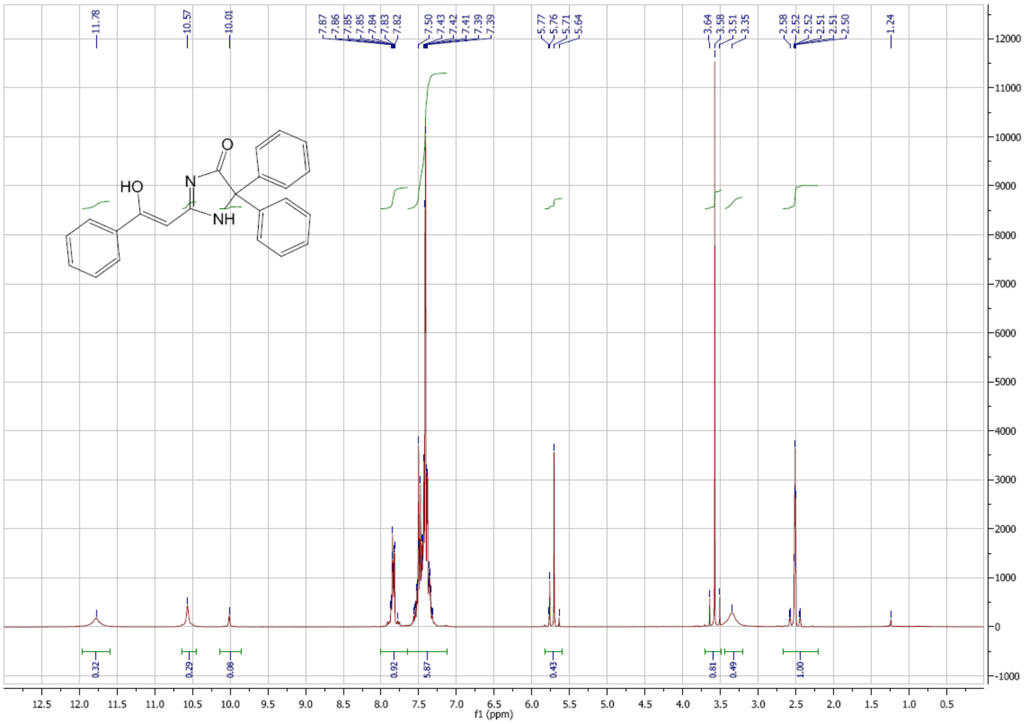

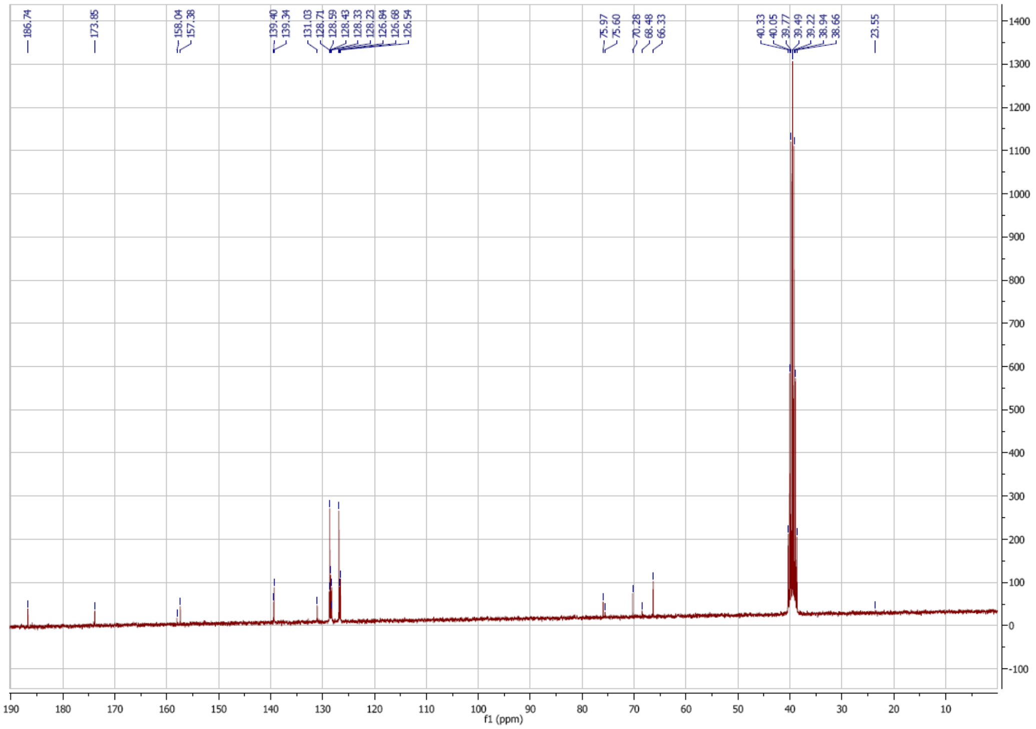

**2-(1*H*-Benzimidazol-2-ylsulfanyl)-1-phenylethanone (3ah)**

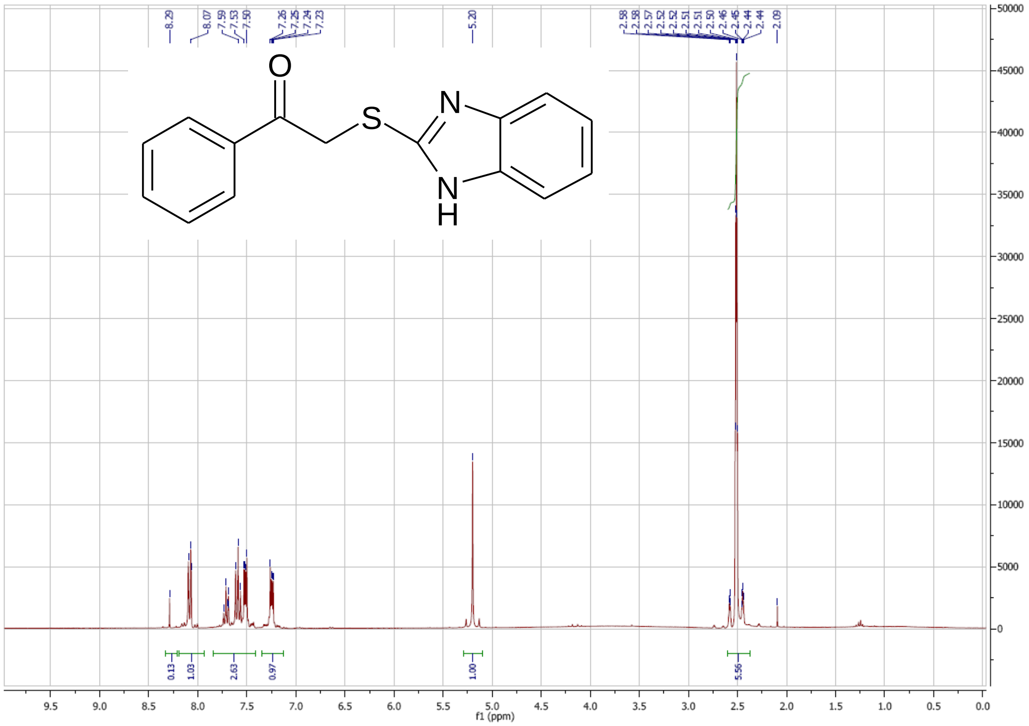


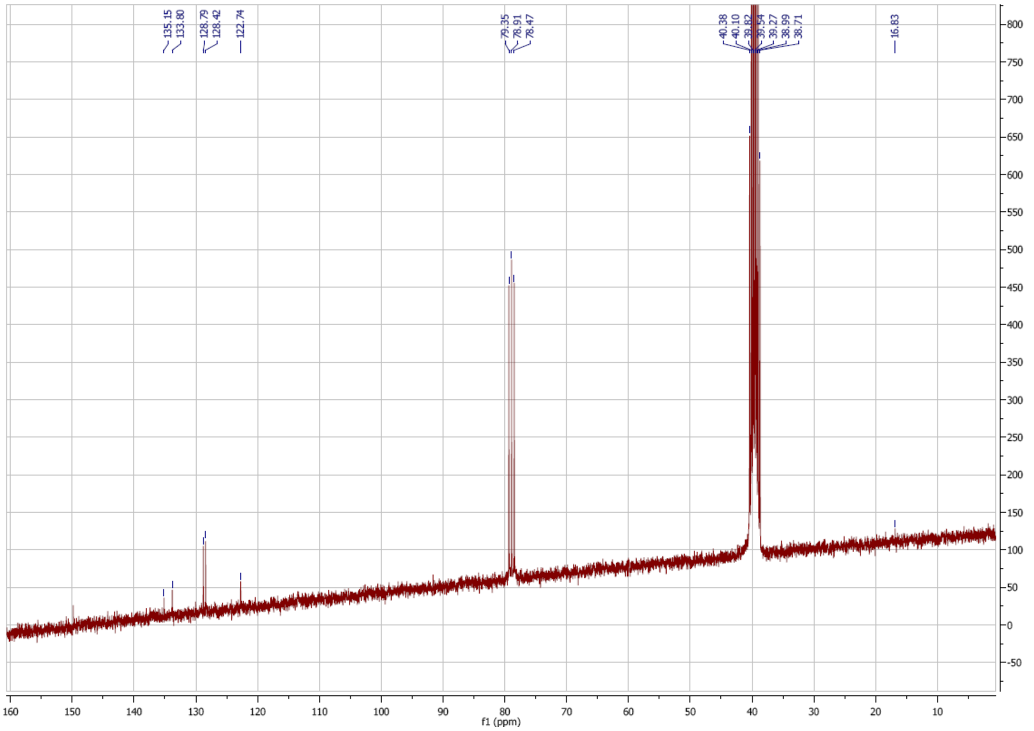

**1-Phenyl-3-[(*E*)-phenylimino]but-1-en-1-ol (4ai)**

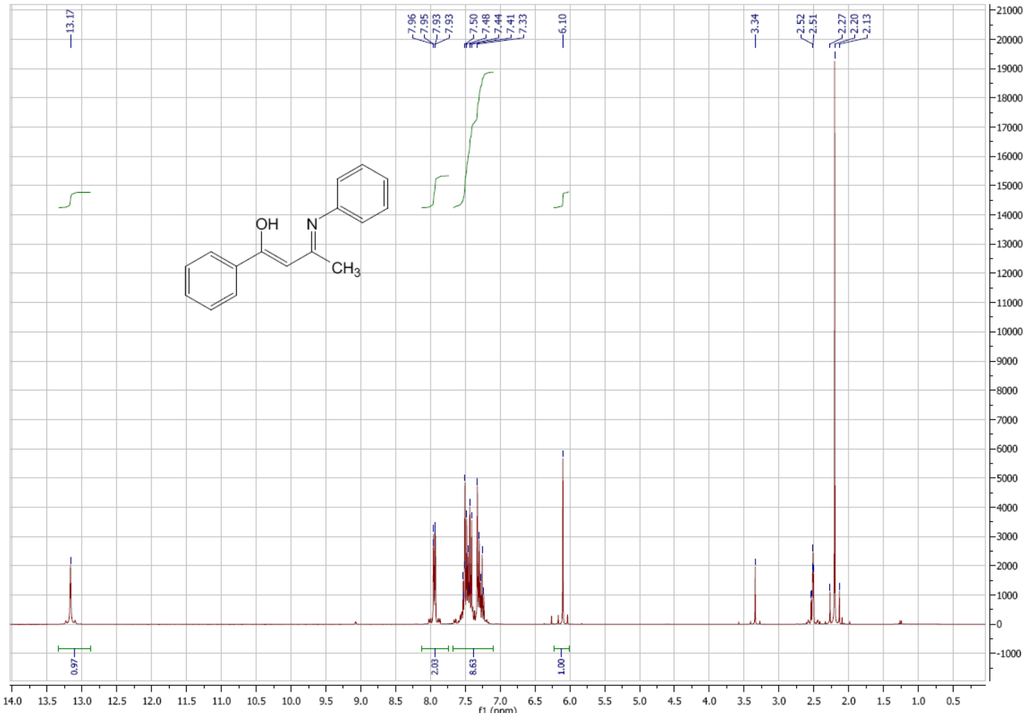


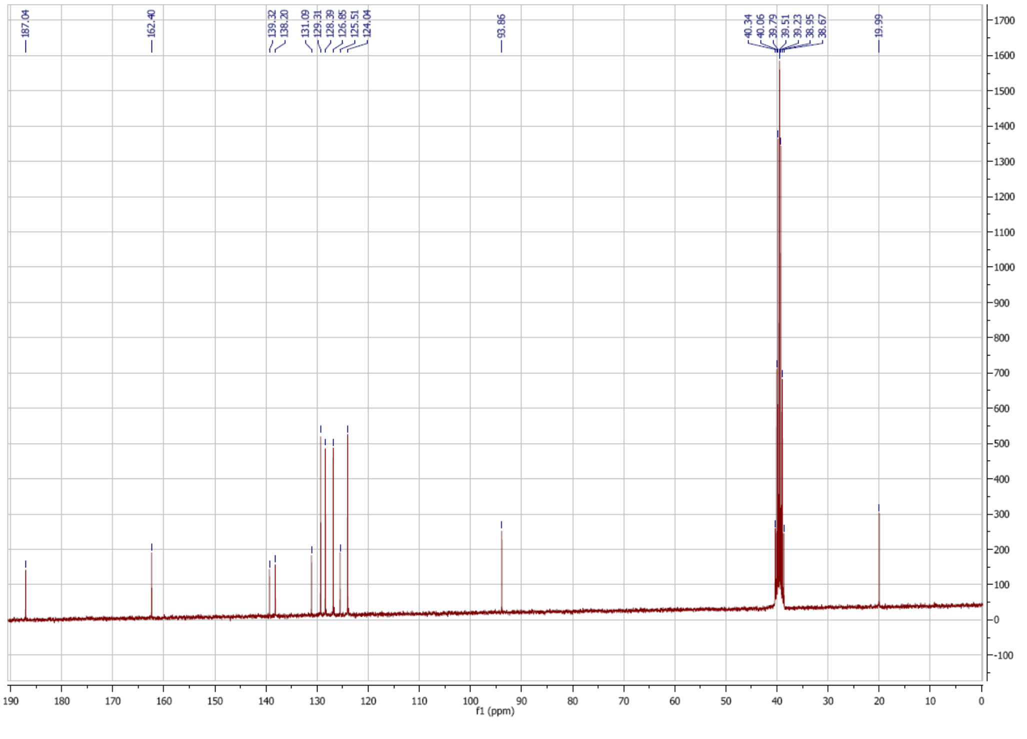

## 2-[2-(4-Bromophenyl)-2-hydroxyvinyl]-6-methylpyrimidin-4-ol (4ba)

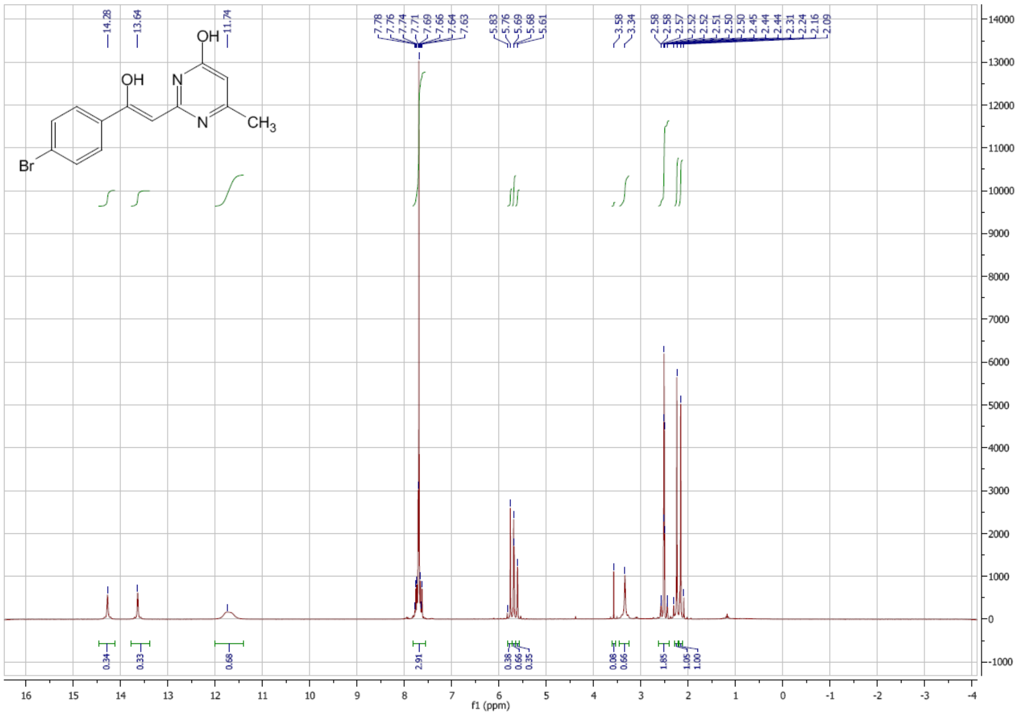


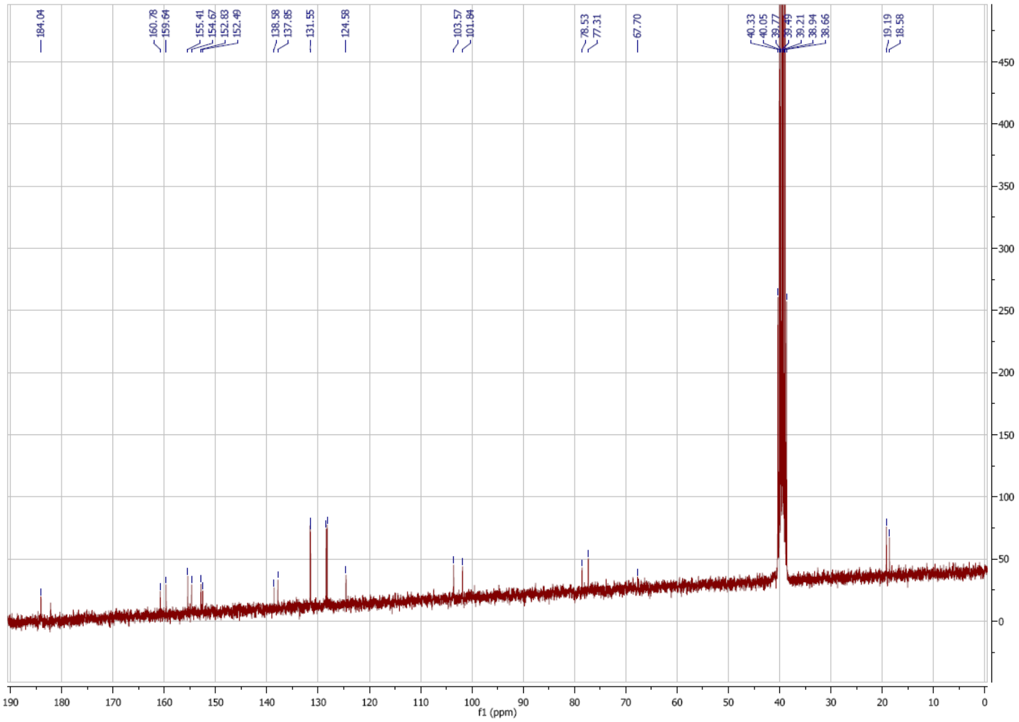

**2-[2-(4-Chlorophenyl)-2-hydroxyvinyl]-6-methylpyrimidin-4-ol (4ca)**

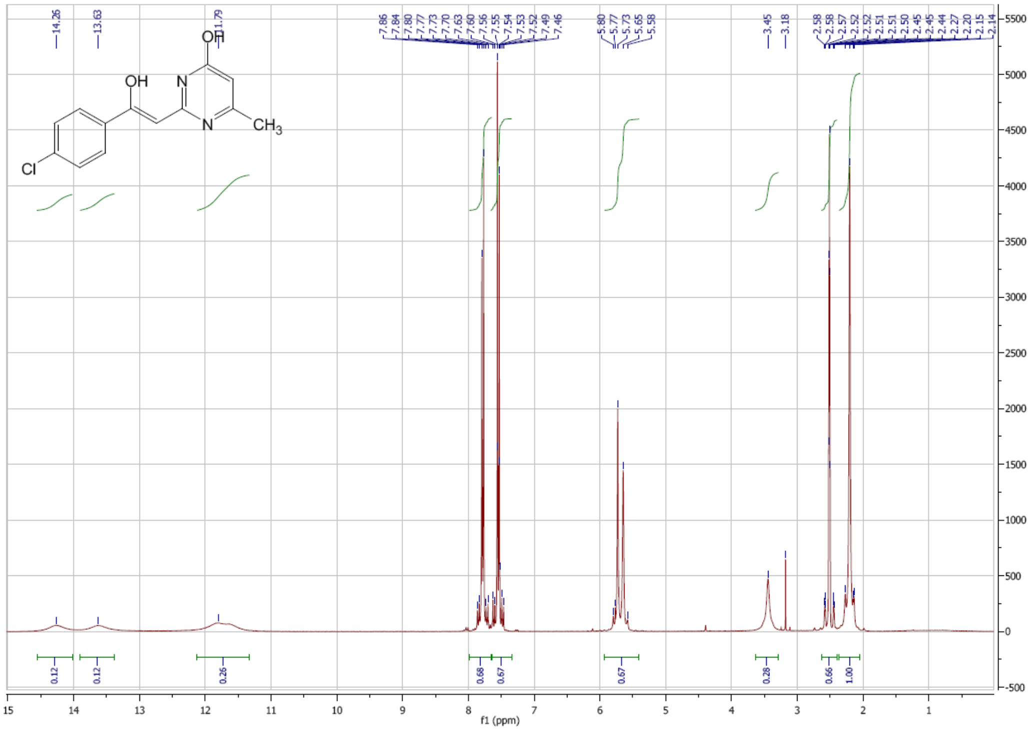


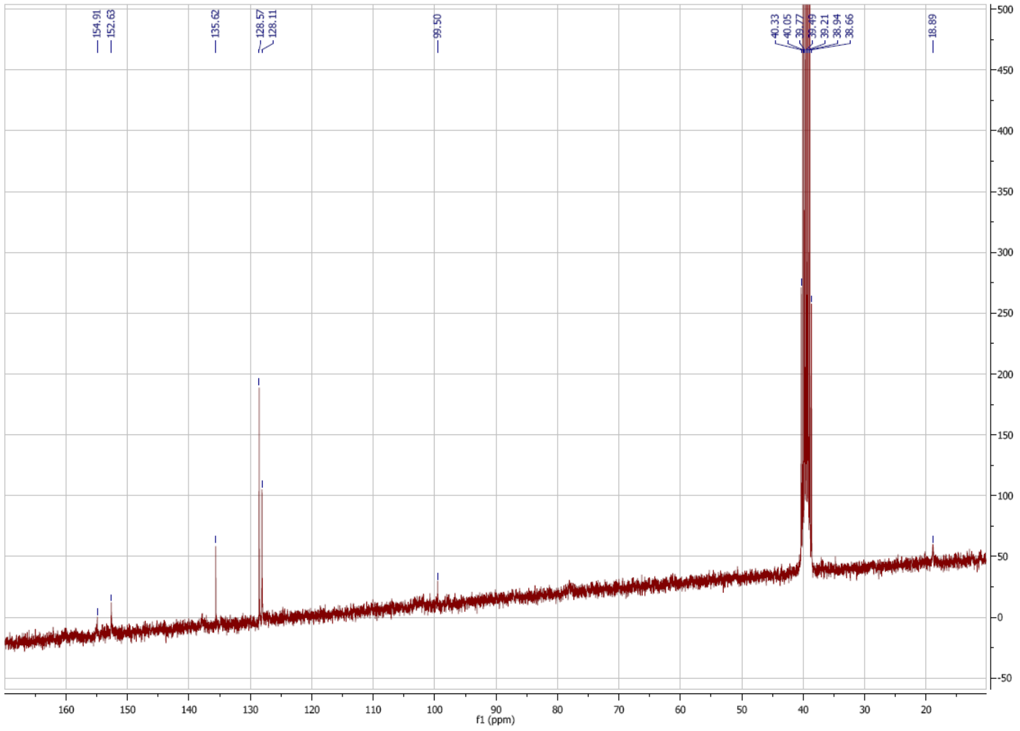

**2-[2-Hydroxy-2-(5-methyl-3-phenylisoxazol-4-yl)vinyl]-6-methylpyrimidin-4-ol (4da)**

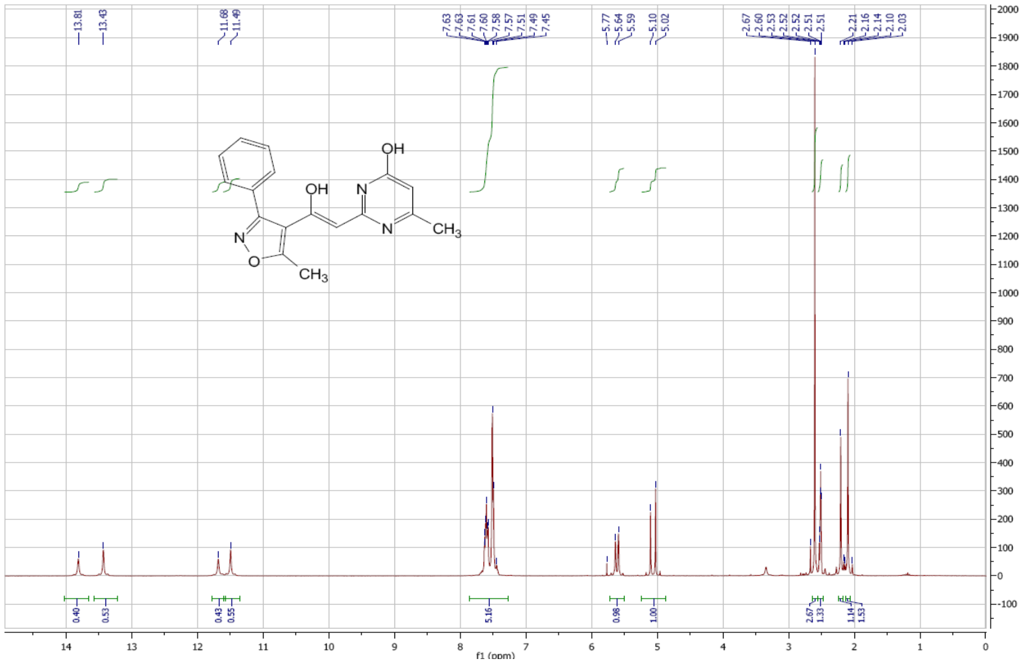

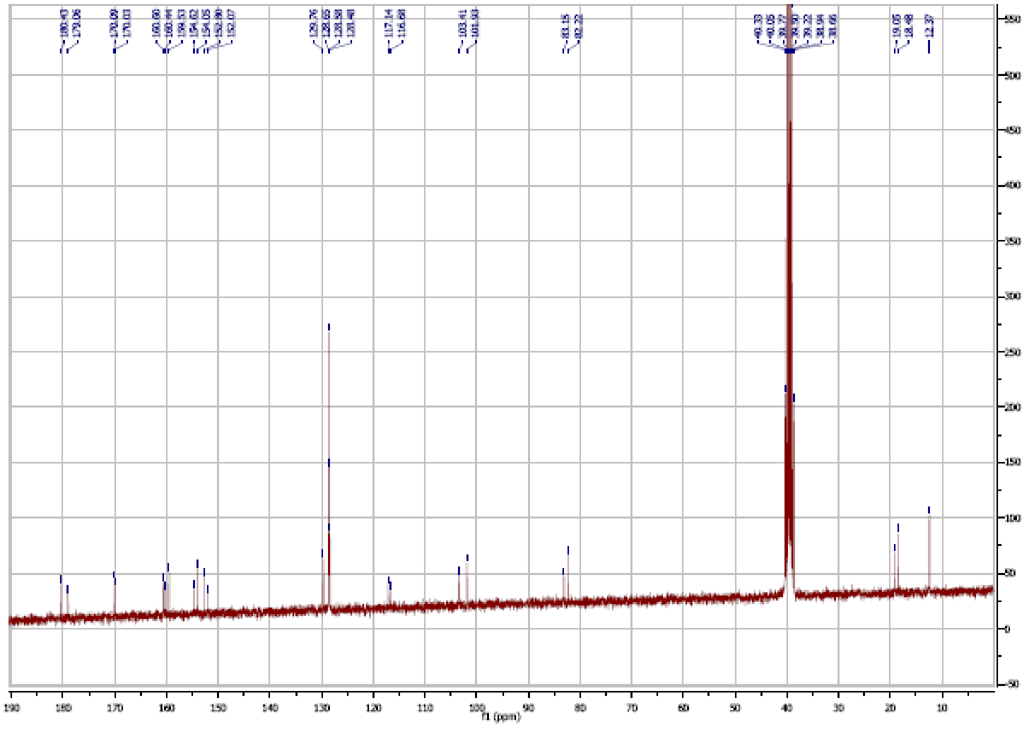

## 2-[(2-Benzofuran-2-yl)-2-hydroxyvinyl]-6-methylpyrimidin-4-ol (4ea)

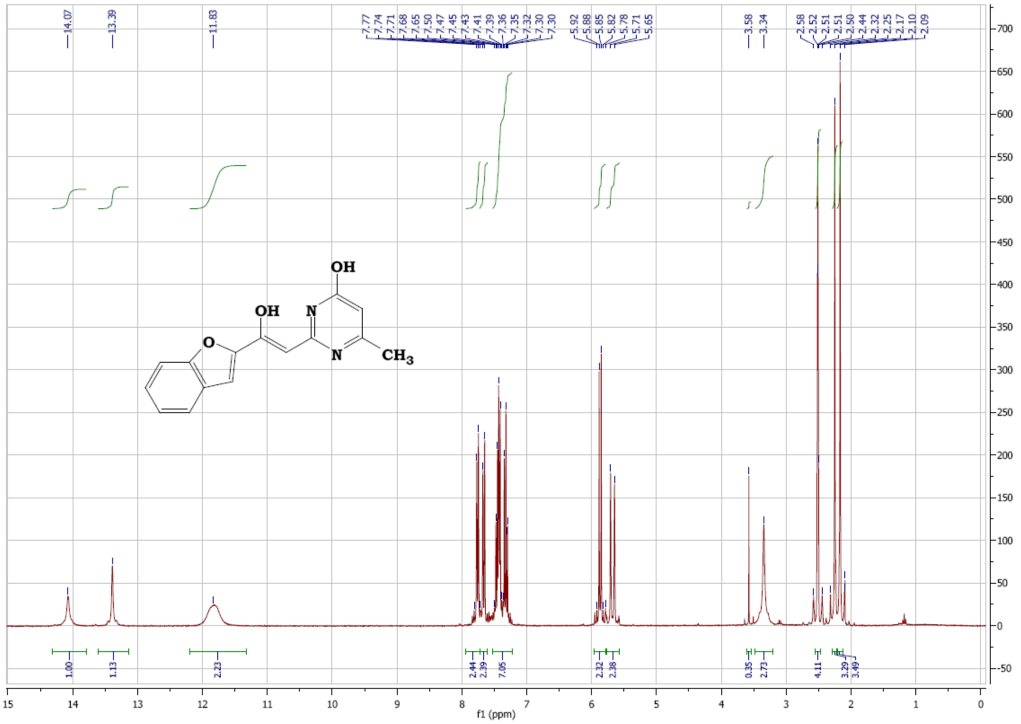

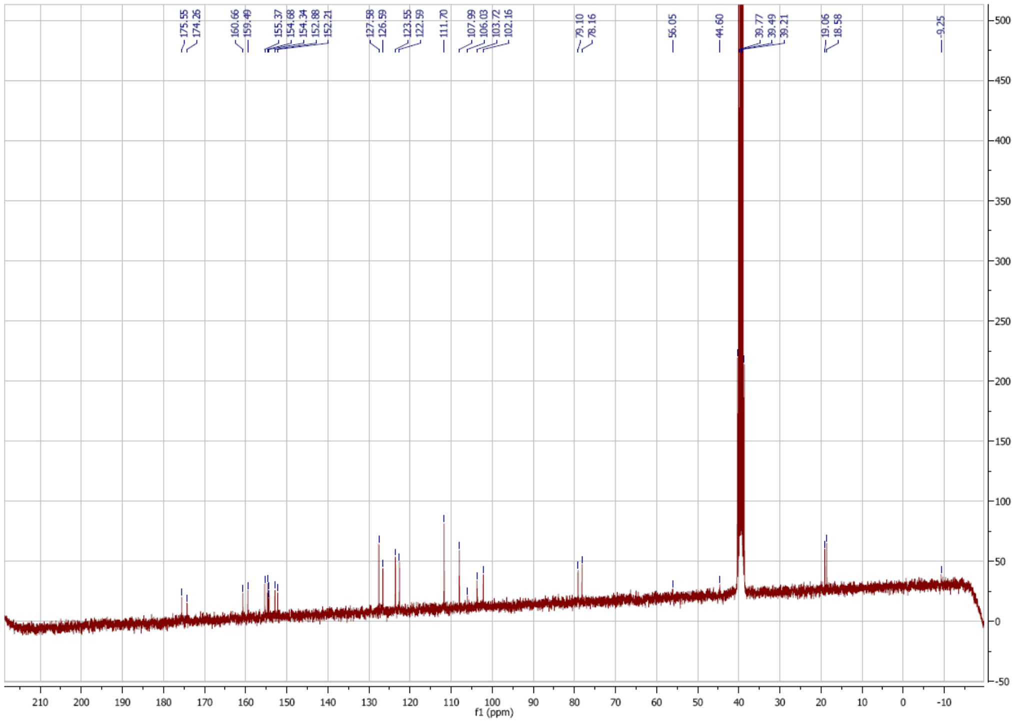

## 2-(2-Hydroxy-2-(naphthalen-2-yl)vinyl)-6-methylpyrimidin-4-ol (4fa)

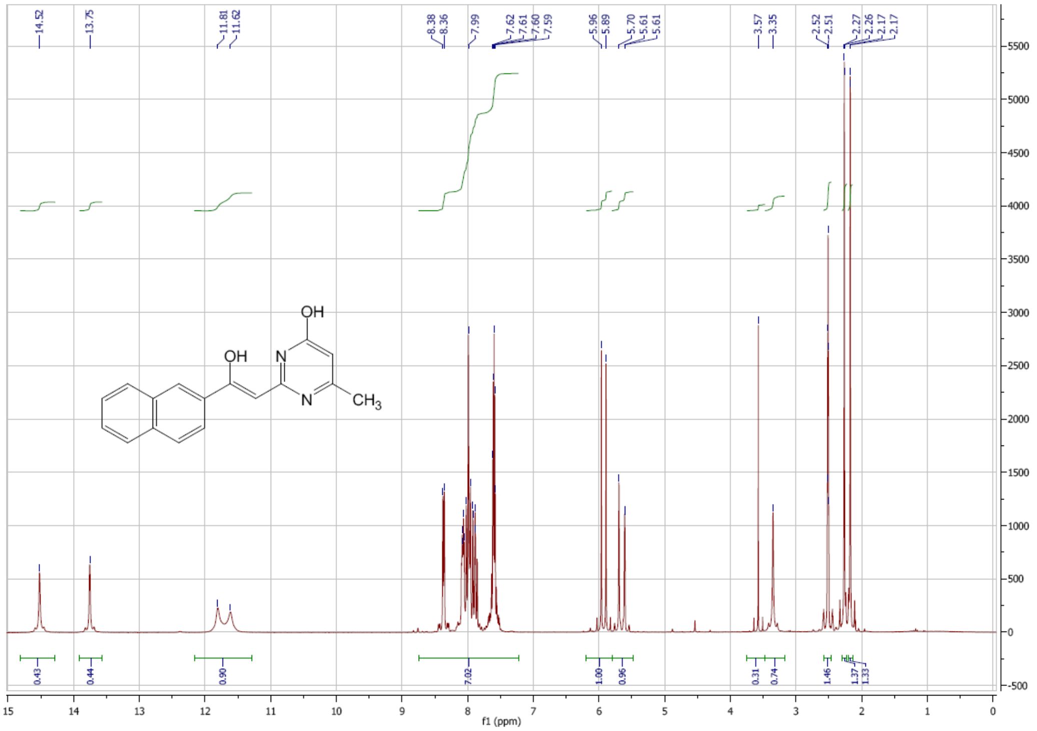


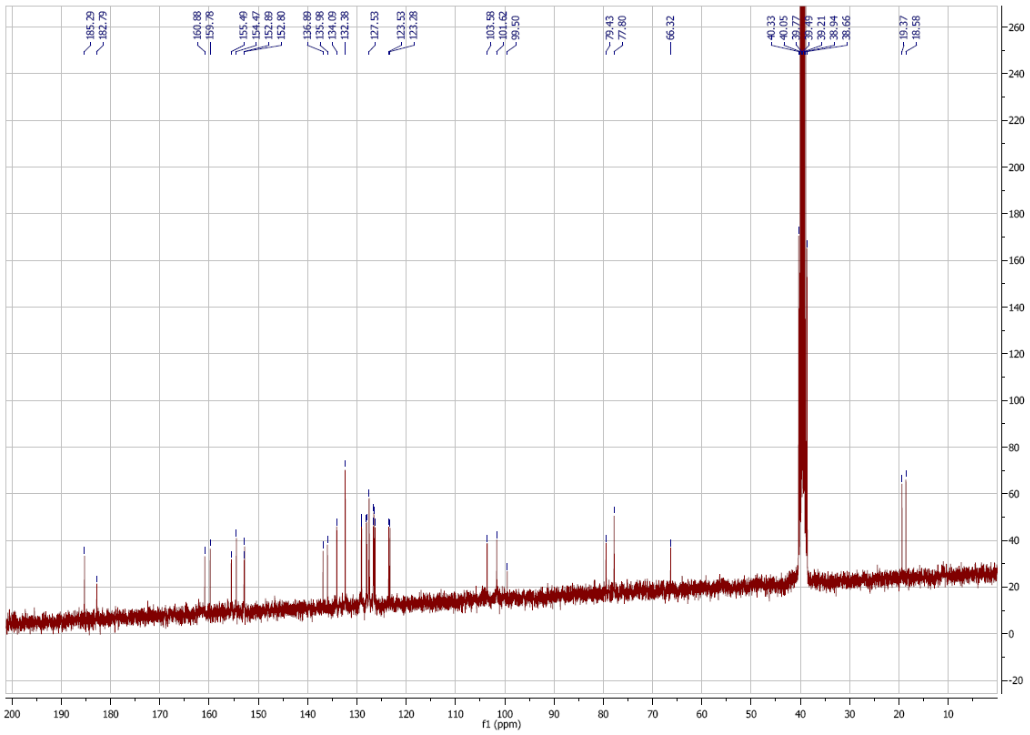

# 2-(2-(1,1'-Biphenyl-4-yl)2-hydroxyvinyl)-6-methylpyrimidin-4-ol (4ga)

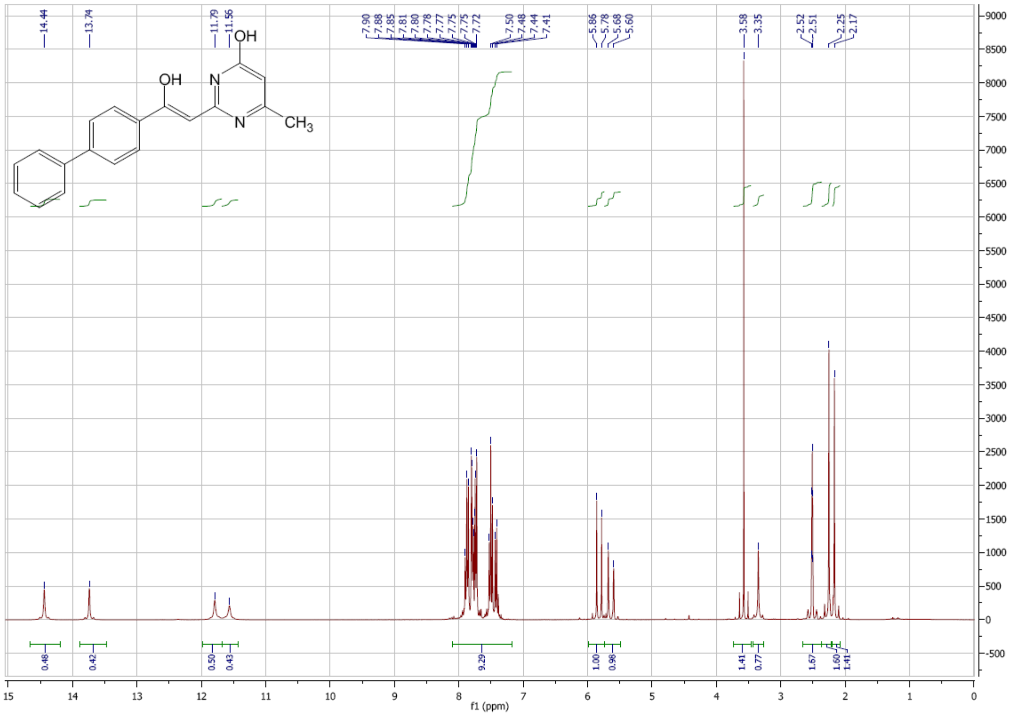


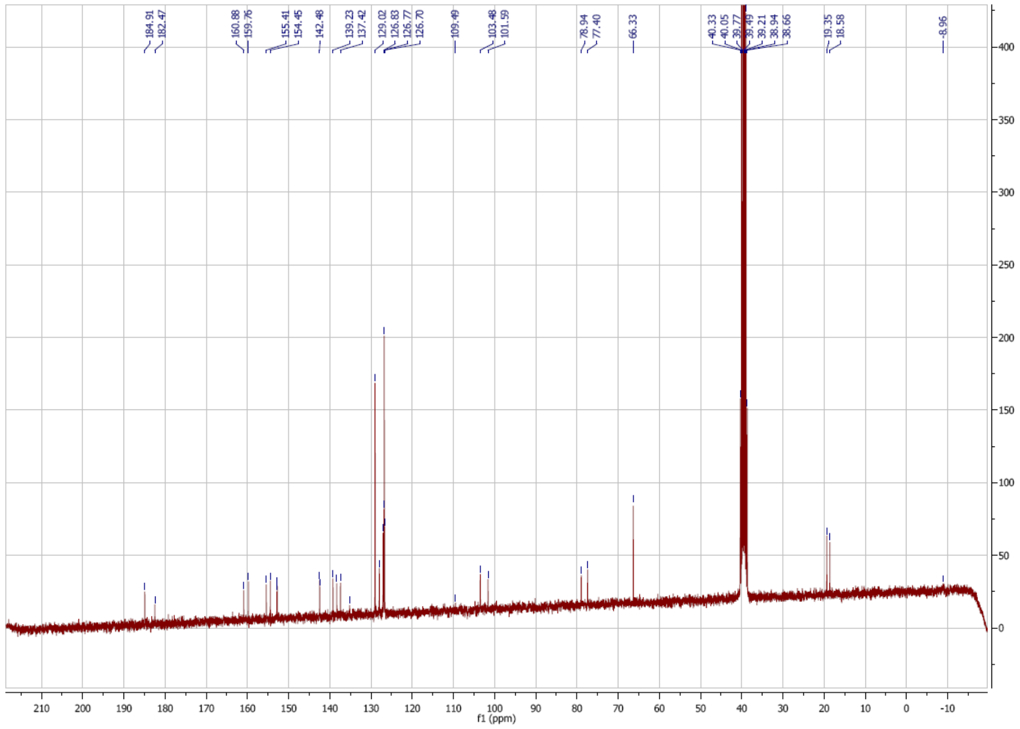

**High resolution mass spectra:**

High resolution mass spectra (HRMS) were recorded on a Bruker Daltonics MicroTOF Focus spectrometer with Na- and Li-formate solutions for internal calibration.

**2-(2-Hydroxy-2-phenylvinyl)-6-methylpyrimidin-4-ol (4aa)**

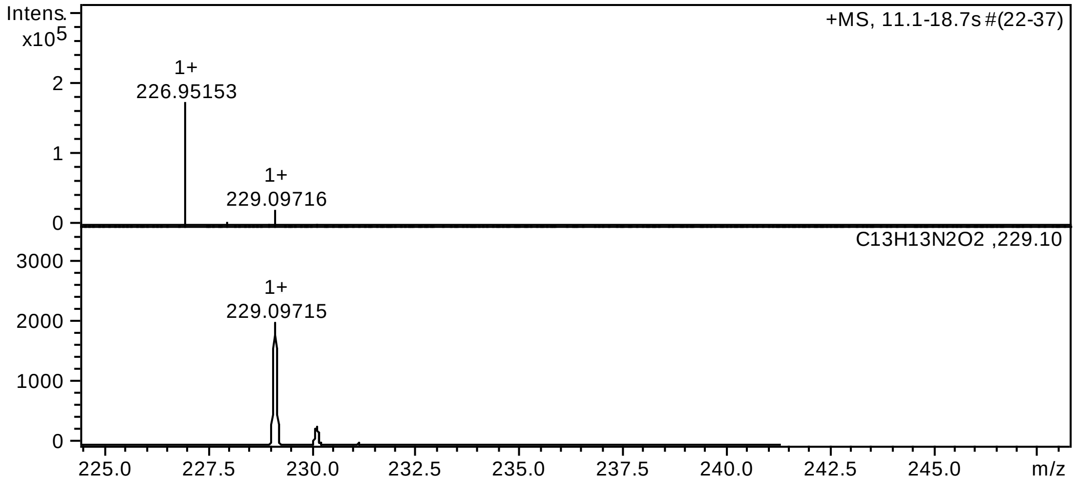


**2-(2-Hydroxy-2-phenylvinyl)-5,6-dimethylpyrimidin-4-ol (4ab)**

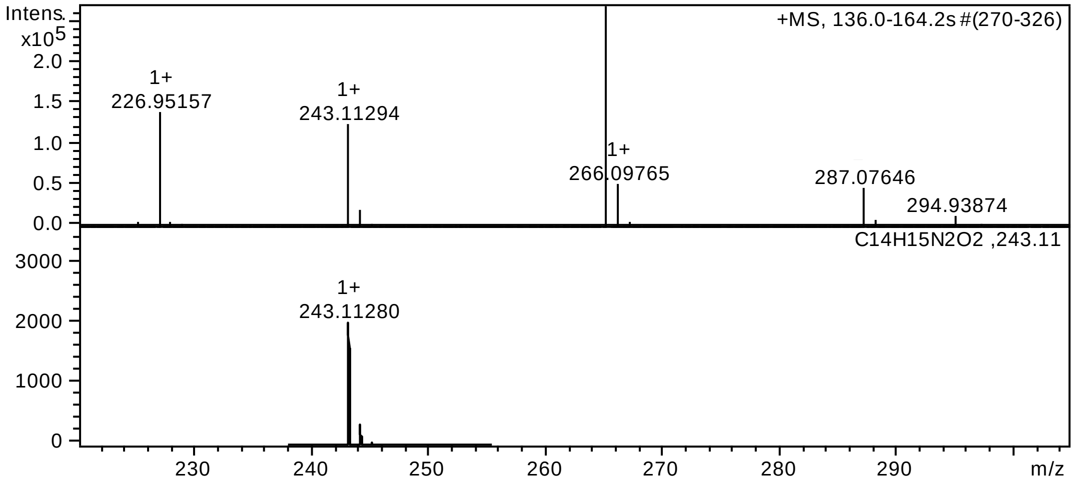

**2-(2-Hydroxy-2-phenylvinyl)quinazolin-4-ol (4ac)**

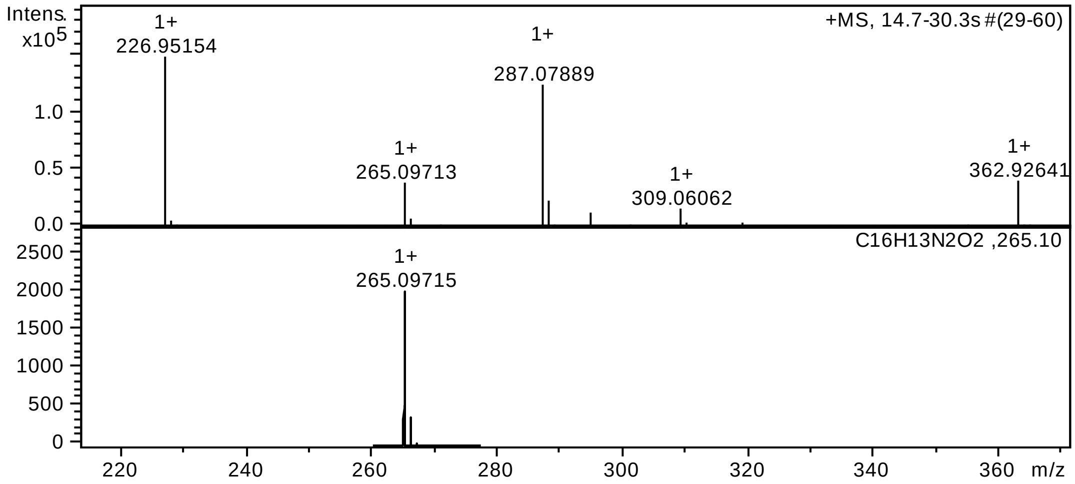


**5-Ethoxycarbonyl-6-methyl-4-(4-methoxyphenyl)-2-(2-hydroxy-2-phenylvinyl)-1,4-dihydropyrimidine (4ae)**

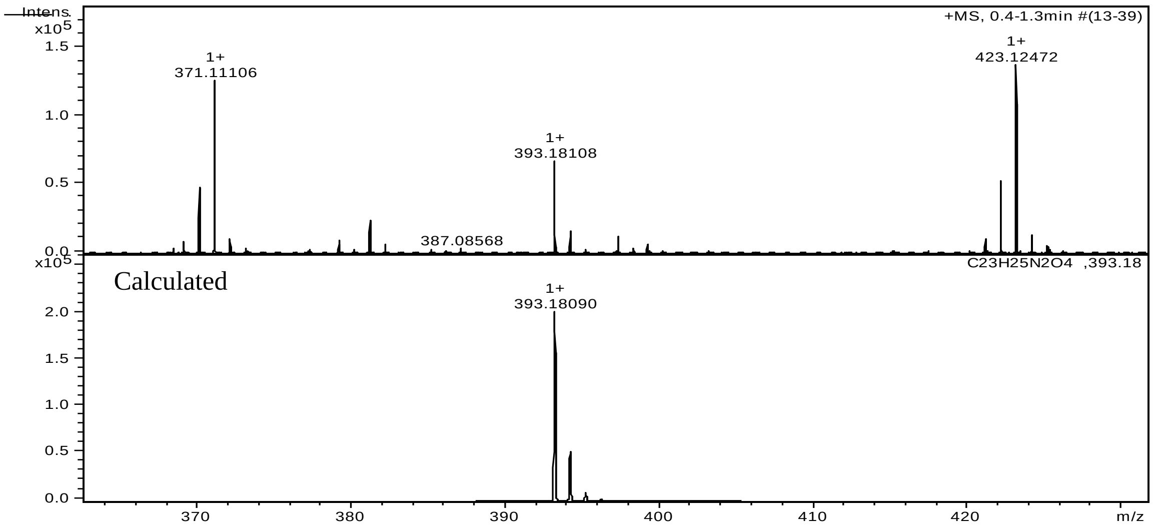

**5-Ethoxycarbonyl-6-methyl-4-phenyl-2-(2-hydroxy-2-phenylvinyl)-1,4-dihydropyrimidine (4af)**

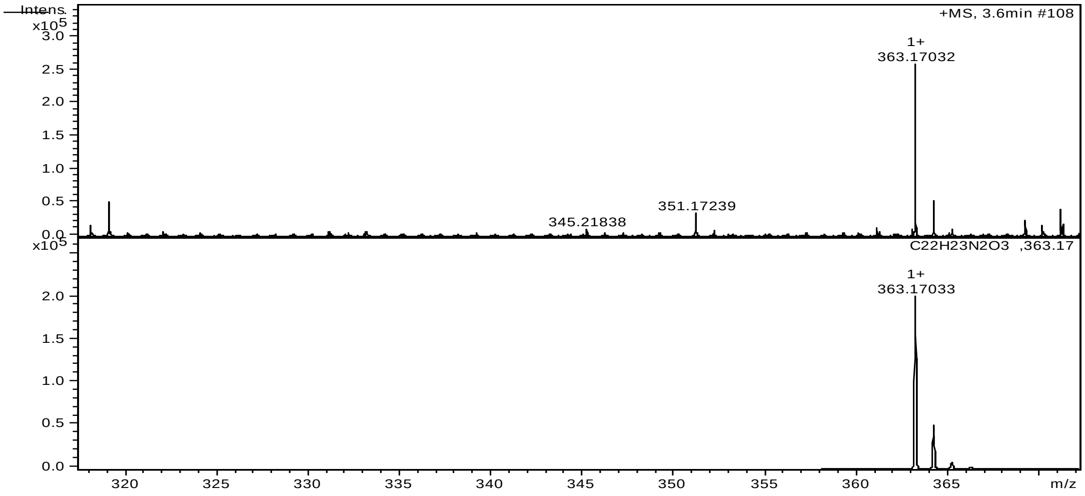


**2-(2-Hydroxy-2-phenylvinyl)-5,5-diphenyl-1,5-dihydroimidazol-4-one (4ag)**

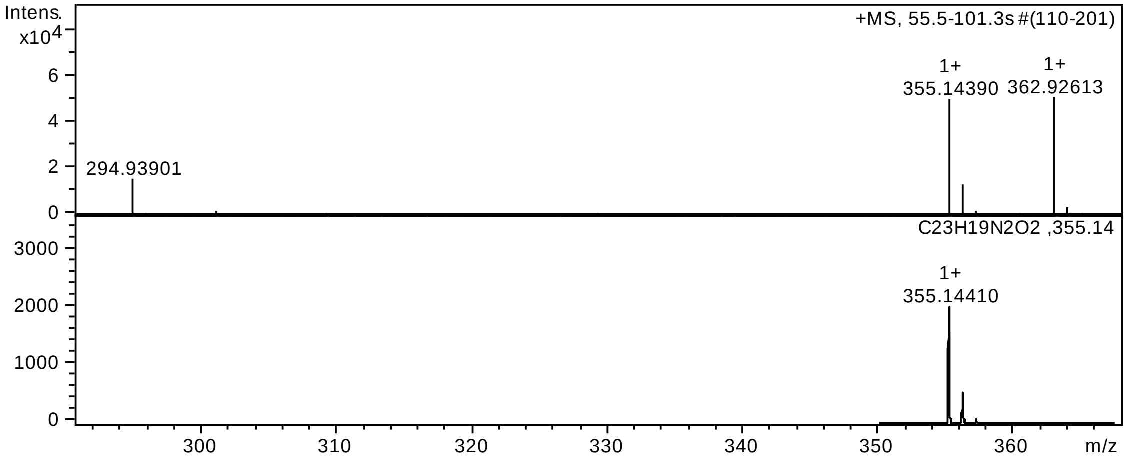

## 2-(1*H*-Benzimidazol-2-ylsulfanyl)-1-phenylethanone (3ah)

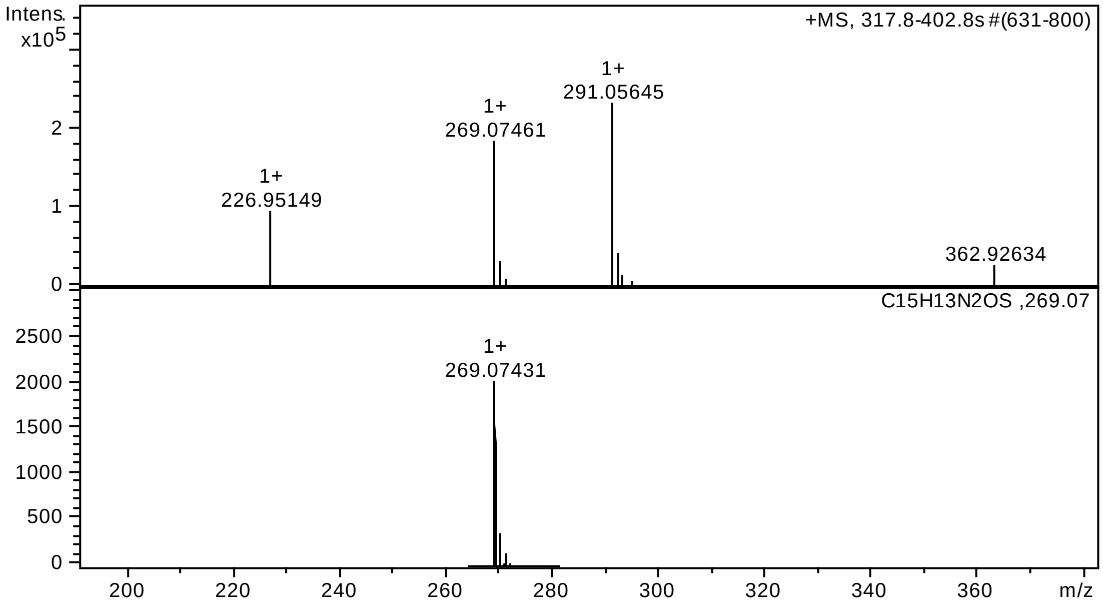


## 1-Phenyl-3-[(*E*)-phenylimino]but-1-en-1-ol (4ai)

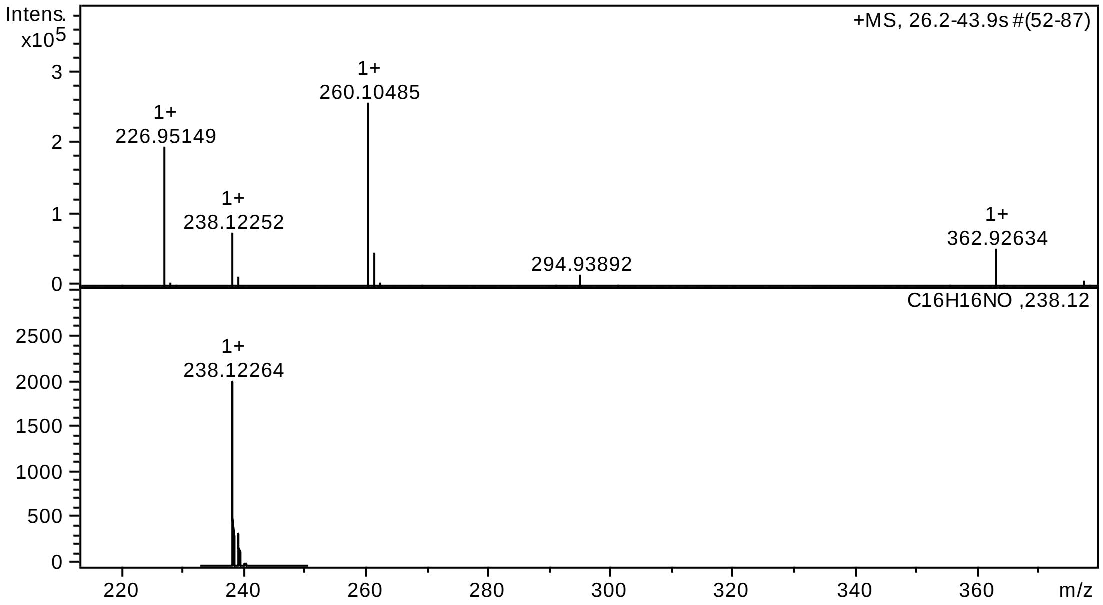

**2-[2-(4-Bromophenyl)-2-hydroxyvinyl]-6-methylpyrimidin-4-ol (4ba)**

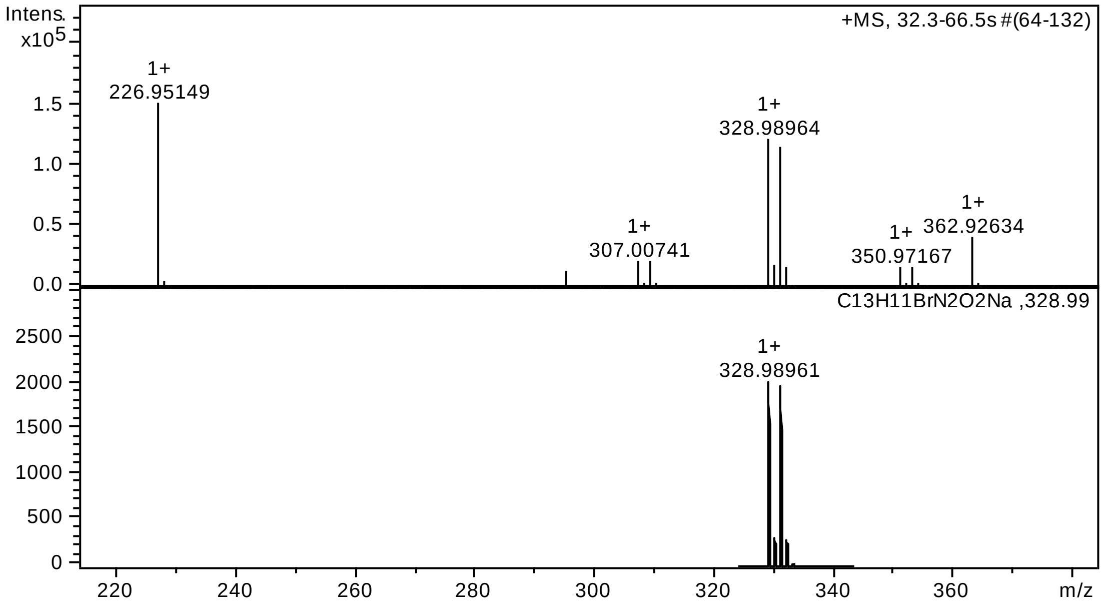


**2-[2-(4-Chlorophenyl)-2-hydroxyvinyl]-6-methylpyrimidin-4-ol (4ca)**

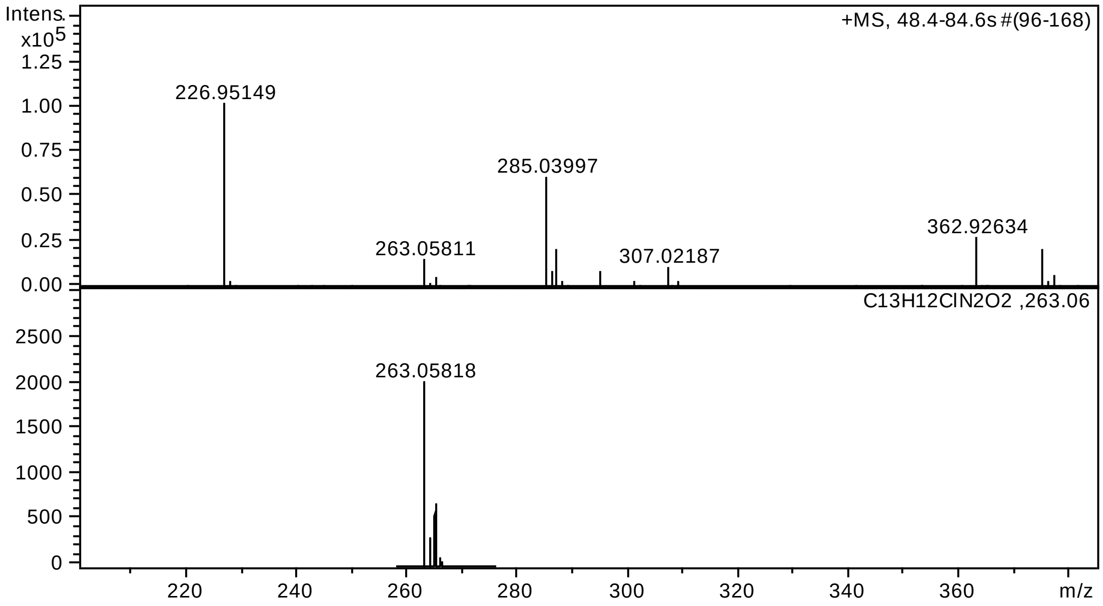

**2-[2-Hydroxy-2-(5-methyl-3-phenylisoxazol-4-yl)vinyl]-6-methylpyrimidin-4-ol (4da)**

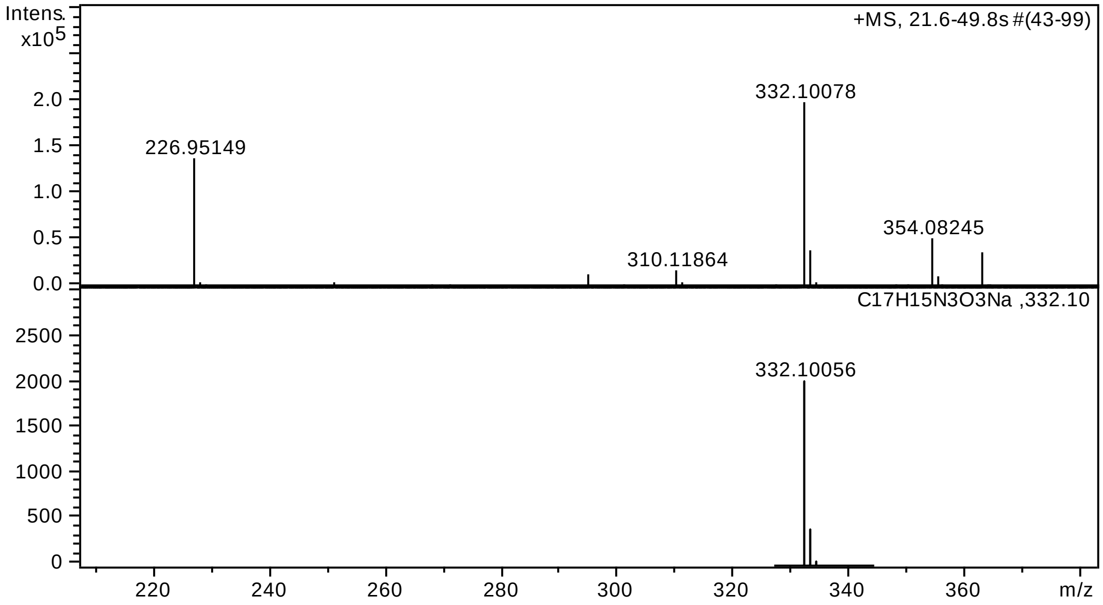


**2-[(2-Benzofuran-2-yl)-2-hydroxyvinyl]-6-methylpyrimidin-4-ol (4ea)**

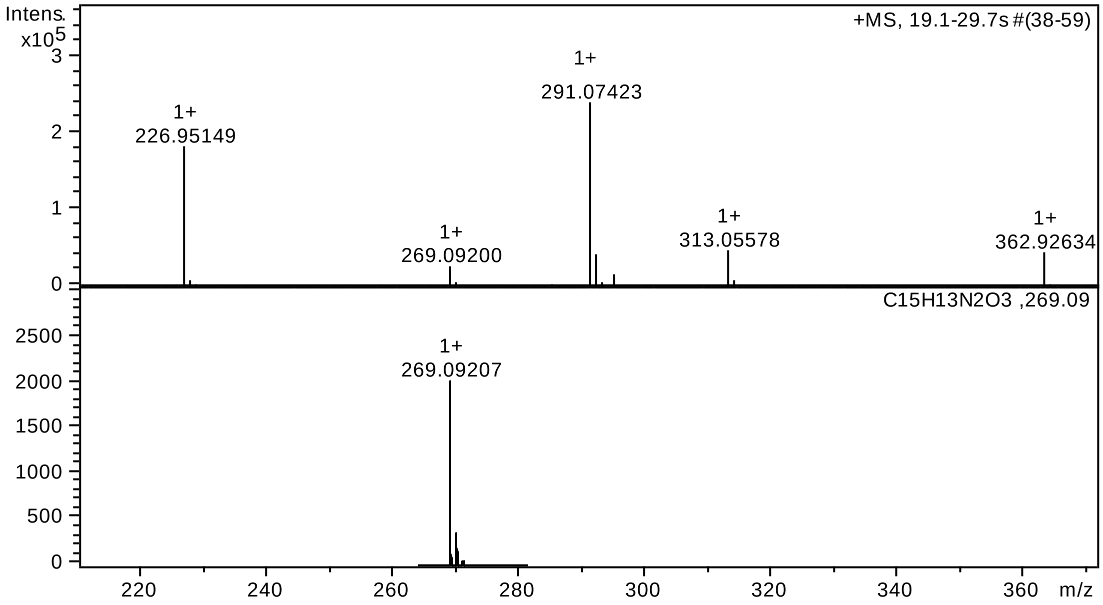

**2-(2-Hydroxy-2-(naphthalen-2-yl)vinyl)-6-methylpyrimidin-4-ol (4fa)**

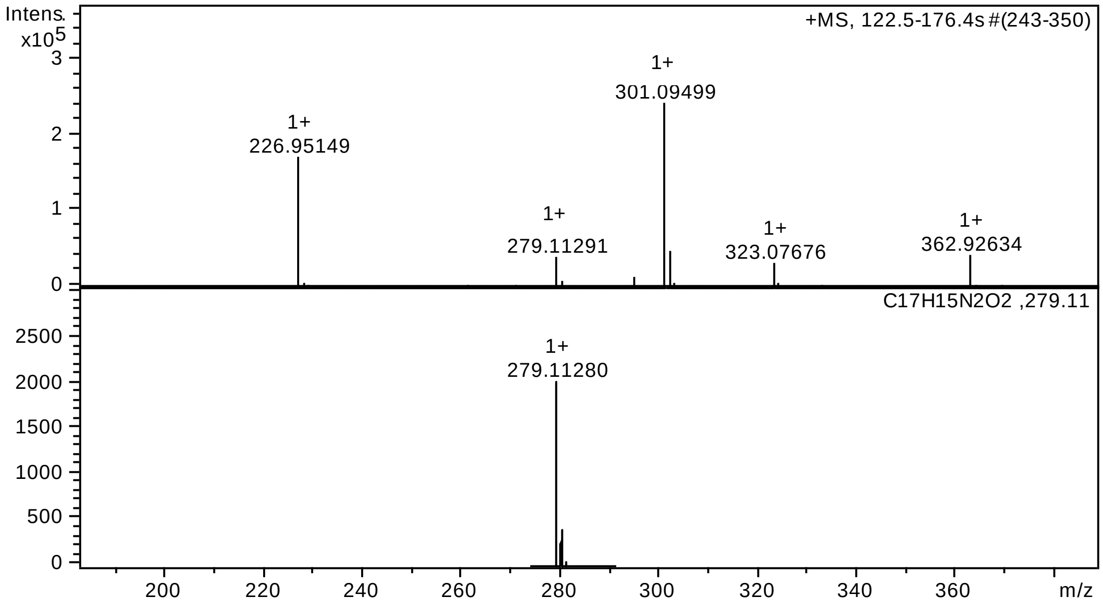


**2-(2-1,1'-Biphenyl-4-yl)2-hydroxyvinyl)-6-methylpyrimidin-4-ol (4ga)**

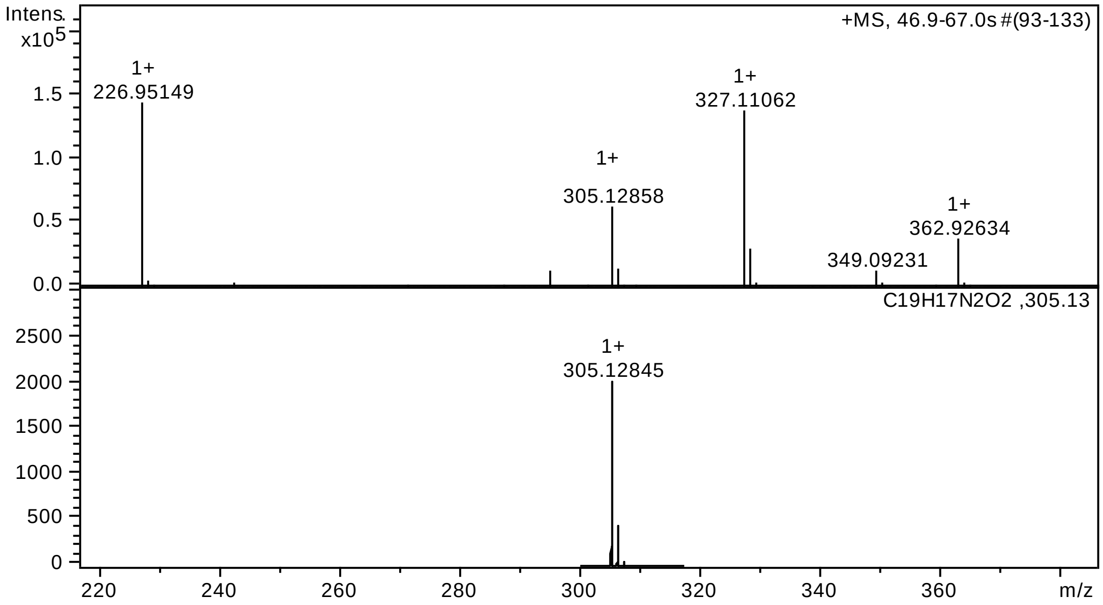

Supplement: File 1 — General procedures, analytical and spectral data. [file Beilstein_J_Org_Chem-07-1164-s001.pdf]
